# Linker histone H1 drives heterochromatin condensation via phase separation in Arabidopsis

**DOI:** 10.1093/plcell/koae034

**Published:** 2024-02-03

**Authors:** Shengbo He, Yiming Yu, Liang Wang, Jingyi Zhang, Zhengyong Bai, Guohong Li, Pilong Li, Xiaoqi Feng

**Affiliations:** Guangdong Laboratory for Lingnan Modern Agriculture, State Key Laboratory for Conservation and Utilization of Subtropical Agro-Bioresources, Guangdong Provincial Key Laboratory of Plant Molecular Breeding, South China Agricultural University, Guangzhou 510642, China; Institute of Science and Technology Austria (ISTA), Am Campus 1, Klosterneuburg 3400, Austria; Institute of Biophysics, Chinese Academy of Science, 15 Datun Road, Chaoyang District, Beijing 100101, China; Beijing Advanced Innovation Center for Structural Biology, Tsinghua University-Peking University Joint Center for Life Sciences, School of Life Sciences, Tsinghua University, Beijing 100084, China; Guangdong Laboratory for Lingnan Modern Agriculture, State Key Laboratory for Conservation and Utilization of Subtropical Agro-Bioresources, Guangdong Provincial Key Laboratory of Plant Molecular Breeding, South China Agricultural University, Guangzhou 510642, China; Guangdong Laboratory for Lingnan Modern Agriculture, State Key Laboratory for Conservation and Utilization of Subtropical Agro-Bioresources, Guangdong Provincial Key Laboratory of Plant Molecular Breeding, South China Agricultural University, Guangzhou 510642, China; Institute of Biophysics, Chinese Academy of Science, 15 Datun Road, Chaoyang District, Beijing 100101, China; Beijing Advanced Innovation Center for Structural Biology, Tsinghua University-Peking University Joint Center for Life Sciences, School of Life Sciences, Tsinghua University, Beijing 100084, China; Institute of Science and Technology Austria (ISTA), Am Campus 1, Klosterneuburg 3400, Austria

## Abstract

In the eukaryotic nucleus, heterochromatin forms highly condensed, visible foci known as heterochromatin foci (HF). These HF are enriched with linker histone H1, a key player in heterochromatin condensation and silencing. However, it is unknown how H1 aggregates HF and condenses heterochromatin. In this study, we established that H1 facilitates heterochromatin condensation by enhancing inter- and intrachromosomal interactions between and within heterochromatic regions of the Arabidopsis (*Arabidopsis thaliana*) genome. We demonstrated that H1 drives HF formation via phase separation, which requires its C-terminal intrinsically disordered region (C-IDR). A truncated H1 lacking the C-IDR fails to form foci or recover HF in the *h1* mutant background, whereas C-IDR with a short stretch of the globular domain (18 out of 71 amino acids) is sufficient to rescue both defects. In addition, C-IDR is essential for H1's roles in regulating nucleosome repeat length and DNA methylation in Arabidopsis, indicating that phase separation capability is required for chromatin functions of H1. Our data suggest that bacterial H1-like proteins, which have been shown to condense DNA, are intrinsically disordered and capable of mediating phase separation. Therefore, we propose that phase separation mediated by H1 or H1-like proteins may represent an ancient mechanism for condensing chromatin and DNA.

## Teaser

Phase separation is a primary mechanism by which linker histone H1 facilitates the condensation of heterochromatin foci and fulfills its role within heterochromatin.

**IN A NUTSHELL**

**Background:** To fit into the nucleus, DNA is densely packaged into structures known as nucleosomes. These nucleosomes consist of core histone proteins around which DNA is wrapped, along with linker histone protein H1, which binds to the DNA between nucleosomes. Parts of the DNA containing repetitive sequences that do not code but can jump around the genome and disrupt genes are further condensed into structures known as heterochromatin foci (HF). These foci appear as visible, dense structures under a microscope, and maintain the silencing of repetitive sequences. H1 proteins are required for the formation of HF and the silencing of repetitive sequences in animals and plants.

**Questions:** How does H1 induce the formation of HF? Is the formation of HF essential for H1's functions in the chromatin?

**Findings:** Our study reveals that H1 condenses heterochromatin in Arabidopsis through a phase separation mechanism. This mechanism refers to the self-aggregating property of macromolecules, leading to the formation of distinct phases. A simple example of this phenomenon is the spontaneous separation of oil and water after mixing. We find that the C-terminal domain (CTD) of H1 mediates chromatin phase separation in vitro and in vivo. Without the CTD, H1 no longer drives the formation of HF or performs normal functions such as regulating nucleosomal repeat length and DNA methylation. Thus, CTD-endorsed phase separation is the primary mechanism by which histone H1 promotes heterochromatin condensation or achieves its function in heterochromatin.

**Next steps:** Our data also suggest that bacterial H1-like proteins, which resemble the CTD of eukaryotic H1 proteins and have been implicated in DNA condensation, can mediate phase separation. Therefore, we propose that phase separation mediated by H1 and H1-like proteins represents an ancient mechanism for compacting chromatin and DNA. Rigorous tests are required to substantiate this idea.

## Introduction

Heterochromatin refers to the highly condensed portion of chromatin, typically forming visible foci that are indicative of its tightly packed structure (Saksouk et al. 2015; Janssen et al. 2018; Montgomery et al. 2020). This portion of chromatin is often abundant in transposable elements (TEs) and is subject to suppression through various epigenetic mechanisms, including DNA methylation, histone modifications (e.g. heterochromatic marks Histone 3 Lysine 9 trimethylation and dimethylation, H3K9me3, and H3K9me2), and histone variants. Frequently enriched in heterochromatic regions, linker histone H1 is a key chromatin structural protein that binds to entry and exit sites of DNA on the surface of nucleosome core particles to stabilize nucleosome structure and condense chromatin (Hergeth and Schneider 2015). H1 plays a crucial role in genome regulation, including the regulation of nucleosomal repeat length (NRL) (Fan et al. 2003; Woodcock et al. 2006; Baldi et al. 2018; Choi et al. 2020) and DNA methylation (Fan et al. 2005; Zemach et al. 2013; Lyons and Zilberman 2017; He et al. 2019; Choi et al. 2020). In Arabidopsis (*Arabidopsis thaliana*) and fruit fly (*Drosophila melanogaster*), H1 preferentially localizes to heterochromatin and is vital for the condensation and formation of heterochromatin foci (HF) (Lu et al. 2009; He et al. 2019; Rutowicz et al. 2019; Choi et al. 2020). Depletion of H1 occurs naturally in the Arabidopsis vegetative cell, and this depletion leads to the complete dispersal of HF and the derepression of TEs (He et al. 2019; He and Feng 2022). However, the precise mechanism by which H1 induces the formation of HF remains unknown.

In Arabidopsis, HF condensation is influenced by various other factors, but their effects are notably weaker compared to H1 mutations. Intact HF is rarely (<3% of the nuclei) observed in the *h1* mutant (as illustrated in Supplementary Fig. S1) (He et al. 2019; Rutowicz et al. 2019; Choi et al. 2020). In contrast, the loss of an H3K9me2 reader, Agenet Domain Containing Protein 1 (ADCP1, also known as AGDP1), or the absence of H3K9me2 writers, SU(VAR)3-9 HOMOLOG 4/5/6 (SUVH4/5/6), abolishes ∼10% to 30% of HF (Stroud et al. 2014; Zhang et al. 2018; Zhao et al. 2019). Mutations of a chromatin remodeler DECREASE IN DNA METHYLATION 1 (DDM1) or DNA methyltransferase METHYLTRANSFERASE 1 (MET1), which drastically reduce cytosine methylation in the genome, induce only a modest degree (∼15% to 30%) of HF decondensation (Stroud et al. 2014; Wang et al. 2017). Similarly, low levels of HF decondensation were observed in mutants of other factors involved in heterochromatin silencing, such as Microrchidia ATPase family protein MORC6, a condensin subunit STRUCTURAL MAINTENANCE OF CHROMOSOME 4 (SMC4), and a histone variant H2A.W (Moissiard et al. 2012; Yelagandula et al. 2014; Wang et al. 2017; Bourguet et al. 2021). Therefore, in Arabidopsis, H1 is the primary factor essential for maintaining HF integrity.

Phase separation has been shown to promote chromatin compartmentalization in both animals and plants (Wang et al. 2023). For instance, histone H2B.8 triggers a distinctive form of euchromatin condensation through phase separation in Arabidopsis sperm (Buttress et al. 2022). H3K9me3/2 readers, such as Heterochromatin Protein 1α (HP1α) and HP1*β* in humans and HP1a in fruit flies, are suggested to condense heterochromatin via promoting phase separation (Larson et al. 2017; Strom et al. 2017; Wang et al. 2019; Wang et al. 2023). The corresponding reader in Arabidopsis, ADCP1, has also been shown to undergo phase separation (Zhao et al. 2019). However, as mentioned above, *adcp1* mutations have a limited effect on HF (Zhao et al. 2019), significantly less than *h1* mutations. Chicken and calf H1 proteins have been shown to promote phase separation of DNA and reconstituted nucleosome arrays (NA) through the C-terminal intrinsically disordered regions (C-IDRs) in vitro, respectively (Gibbs and Kriwacki 2018; Turner et al. 2018; Gibson et al. 2019). However, whether and how C-IDRs contribute to the phase separation capability of H1 in vivo has not been tested. Furthermore, it remains unknown if phase separation is a primary mechanism by which histone H1 promotes HF condensation or achieves its function in heterochromatin.

Here, we show that H1 condenses heterochromatin through a phase separation mechanism in Arabidopsis. We demonstrate that the C-IDR of H1 mediates its phase separation, which is crucial for H1's preferential deposition into heterochromatic regions and its regulatory roles in controlling NRL and DNA methylation. Moreover, our data indicate that bacterial H1-like proteins are intrinsically disordered and can mediate phase separation, resembling the C-IDRs of eukaryotic H1 proteins. This observation suggests that the phase separation of H1 and H1-like proteins may constitute an ancient mechanism for compacting chromatin and DNA.

## Results

### Higher-order chromatin architecture is altered in the *h1* mutant

In Arabidopsis, there are three H1 paralogs: H1.1 and H1.2, two major paralogs with a high degree of sequence similarity and overlapping functions in chromatin compaction, which are ubiquitously expressed in most tissues, and H1.3, which is specifically activated in response to stress conditions (Rutowicz et al. 2015). Using the *h1.1h1.2* double mutant (referred to as the *h1* mutant hereafter), in which intact HF is rarely (<3% of the nuclei) observed (He et al. 2019; Rutowicz et al. 2019; Choi et al. 2020), we performed genome-wide chromosome conformation capture analysis (Hi-C) to understand how H1 affects higher-order chromatin architecture. In total, we obtained 177 and 189 million valid interactions for the wild-type (WT) and *h1* mutant, respectively, and *cis* (intrachromosomal) interactions account for nearly 80% of total valid interactions (Supplementary Fig. S2). The high enrichment of *cis* interactions and the resemblance between our Hi-C maps (Supplementary Fig. S3) and those previously published (Feng et al. 2014; Grob et al. 2014; Wang et al. 2015; Liu et al. 2016; Hu et al. 2019; Teano et al. 2023) validate the quality of our datasets.

**Figure 1.**
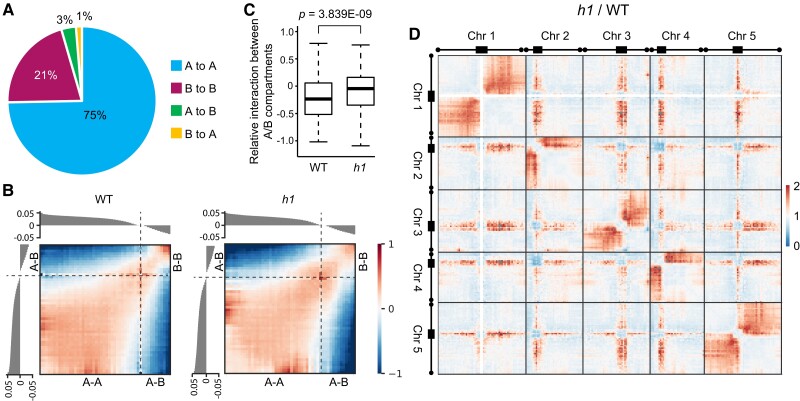
*H1* mutations cause increased interactions between heterochromatin and euchromatin and decreased interactions within heterochromatin. **A)** Pie chart showing the shifting of A/B compartments in *h1* mutant relative to WT at 25-kb resolution. The ratios of each part are shown. **B)** Saddle plots of chromatin compartmentalization depicting normalized interactions between and within A/B compartments at 25-kb resolution in the indicated genotypes. The average compartment scores of 50 percentiles at 25-kb resolution are ordered and shown above. **C)** Box plots illustrating relative interactions between A/B compartments at 25-kb resolution in the indicated genotypes. Each box encloses the middle 50% of the distribution, with the horizontal line marking the median and vertical lines marking a 1.5× interquartile range (IQR) or the minimum and maximum values that fall within 1.5× IQR. Mann–Whitney *U*-test *P* value is shown above. **D)** Hi-C map showing the difference of interactions between *h1* mutant and WT at 500-kb resolution.

Chromatin is typically structured into two distinct compartments known as A and B compartments, which predominantly correspond to active/euchromatic and inactive/heterochromatic domains, respectively (Feng et al. 2014; Grob et al. 2014). Our Hi-C data show that the A and B compartments account for similar percentages between the WT and *h1* mutant, with only marginal A/B conversions occurring (Fig. 1A), indicating compartmentalization is largely maintained in the *h1* mutant. However, interactions between A/B compartments are significantly enhanced in the *h1* mutant compared to in the WT (Fig. 1, B and C). This is most obvious in pericentromeric regions, whose interactions with chromosome arms are enhanced both within and across chromosomes in the mutant. A corresponding depletion of interactions is also evident both within and between pericentromeric regions of chromosomes (Fig. 1D). These data indicate that pericentromeric heterochromatin is decondensed in the *h1* mutant, consistent with cytological observations (He et al. 2019; Rutowicz et al. 2019; Choi et al. 2020) and a previous report (Teano et al. 2023). Likely as a result, the pericentromere is no longer able to hinder contact between the two arms of each chromosome (Berr and Schubert 2007; Schubert et al. 2012; Grob et al. 2013; Feng et al. 2014), as denoted by the enhanced interactions between the two arms in the *h1* mutant compared to in the WT (Fig. 1D). These findings indicate substantial impacts of H1 on the higher-order organization of chromatin.

### H1 undergoes phase separation in a DNA/NA-dependent manner

**Figure 2.**
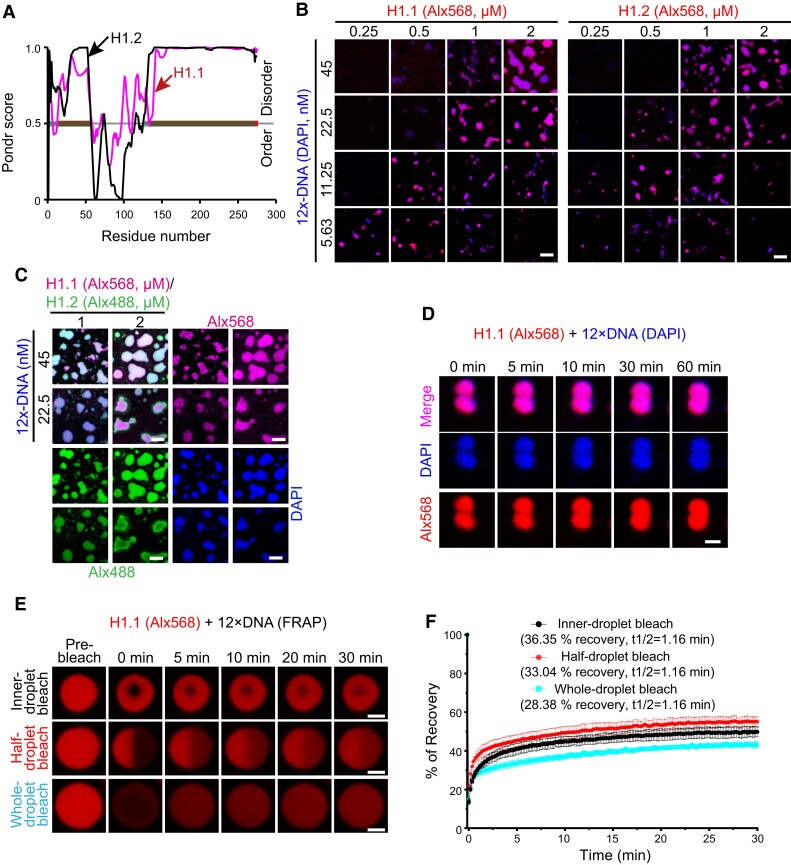
H1 undergoes DNA-dependent phase separation in vitro. **A)** Predictor of natural disordered regions (PONDR) score for H1. A score > 0.5 is considered disordered. **B)** Phase diagrams of H1.1 and H1.2 with 12 × Widom 601 DNA. The concentrations are indicated. **C)** Phase diagram showing H1.1 and H1.2 mix in vitro when H1.1 was added to pre-existing H1.2 puncta after incubating overnight. **D)** In vitro H1.1-DNA condensate fusion over time. **E, F)** FRAP on the puncta formed by H1.1 with 12 × DNA in vitro. Recoveries are shown in the plot (F). Data are presented as mean ± s.d. (*n* = 6). Recovery rates and the time (t1/2) required for half recovery are indicated. Droplets are inner-, half-, or whole-bleached, respectively. H1.1/H1.2 is labeled with Alx568/Alx488 and DNA is stained with DAPI in (B-E). The merged channel is shown in (B). The Alx568 channel is shown in (E). All channels are shown in (C, D). Scale bars, 5 *μ*m (B, C) and 2 *μ*m (D, E).

Both Arabidopsis H1.1 and H1.2 are predicted to have IDRs at their N (∼57 aa for H1.1 and ∼55 aa for H1.2) and C (∼146 aa for H1.1 and ∼149 aa for H1.2) termini (Fig. 2A). Also, both H1 proteins form nuclear bodies colocalizing with HF (He et al. 2019). We therefore hypothesized that H1 drives heterochromatin domain formation through a phase separation mechanism. To test this, we performed an in vitro phase separation assay and found that H1.1 and H1.2 cannot phase separate on their own even under high concentrations (e.g. 15 *μ*M; Supplementary Fig. S4). In contrast, the addition of DNA induces puncta formation, with as low as 0.25 *μ*M of H1 forming condensates with DNA (Fig. 2B). Phase separation of H1-DNA condensates occurs in a concentration-dependent manner (Fig. 2B), indicating specific ratios of H1 to DNA are required.

In Arabidopsis, H1.1 and H1.2 exhibit overlapping roles in maintaining heterochromatin condensation and are deposited to similar genomic regions (He et al. 2019; Rutowicz et al. 2019; Choi et al. 2020). When H1.1 is added to pre-existing H1.2/DNA condensates or *vice versa*, all three of which are tagged with different fluorophores, condensates become homogenously triple-labeled over a prolonged period (Fig. 2C and Supplementary Fig. S5). This observation shows that the two H1 isoforms can coexist and intermingle within the same phase-separated condensates. This result aligns with the overlapping functions of H1.1 and H1.2 *in planta* and their substantial sequence similarities and shared expression patterns (Zemach et al. 2013; Rutowicz et al. 2015; Rutowicz et al. 2019).

We next examined the physical properties of H1 condensates. H1 puncta exhibit a slow merging pattern upon contact, which does not achieve complete fusion even after 60 min (Fig. 2D), suggesting a gel-like property. We subsequently performed fluorescence recovery after photobleaching (FRAP) experiments with individual puncta that were subjected to partial, half, or complete bleaching. Consistent with the gel-like property of H1 puncta, we observed consistently low levels of recovery (∼28% to ∼36%) with comparable half-recovery rates (t_1/2_ = 1.16 min), regardless of the extent of puncta bleaching (Fig. 2E).

**Figure 3.**
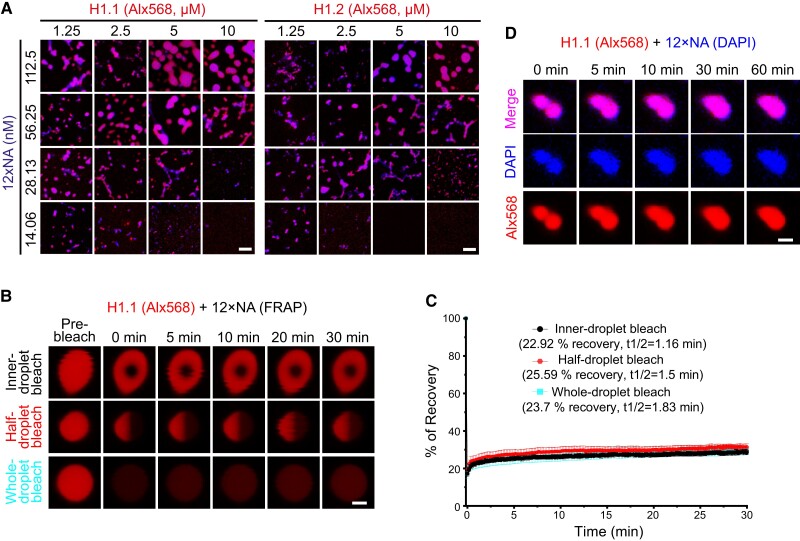
H1 undergoes NA-dependent phase separation in vitro. **A)** Phase diagrams of H1.1 and H1.2 with 12 × nucleosome array (NA). The concentrations are indicated. **B, C)** FRAP on the puncta formed by H1.1 with 12 × NA in vitro. Recoveries are shown in the plot (C). Data are presented as mean ± s.d. (*n* = 6). Recovery rates and the time (t1/2) required for half recovery are indicated. Droplets are inner-, half-, or whole-bleached, respectively. **D)** In vitro H1.1-NA condensate fusion over time. H1.1/H1.2 are labeled with Alx568 and DNA is stained with DAPI in (A, B, and D). The merged channel is shown in (A). The Alx568 channel is shown in (B). All channels are shown in (D). Scale bars, 5 *μ*m (A) and 2 *μ*m (B, D).

We further reconstituted NA using recombinant Arabidopsis core histones and observed similar puncta formation in the presence of H1 (Fig. 3A). Moreover, FRAP experiments with partial, half, or complete bleaching of H1.1 puncta in the context of NA show similarly low levels (∼23% to ∼25.6%) of recovery and comparable half-recovery rates (t_1/2_ = 1.16 min for partial, 1.5 min for half, and 1.83 min for complete bleaching; Fig. 3, B and C) compared to in the context of DNA (Fig. 2, E and F), suggesting the gel-like nature of H1-mediated nucleosome phase separation. Consistently, NA-based H1.1 puncta show gradual and incomplete fusion over an extended duration (Fig. 3D). Taken together, our data indicate that H1 undergoes gel-like phase separation in the presence of DNA/NA.

### H1-enriched HF are gel-like condensates in vivo

**Figure 4.**
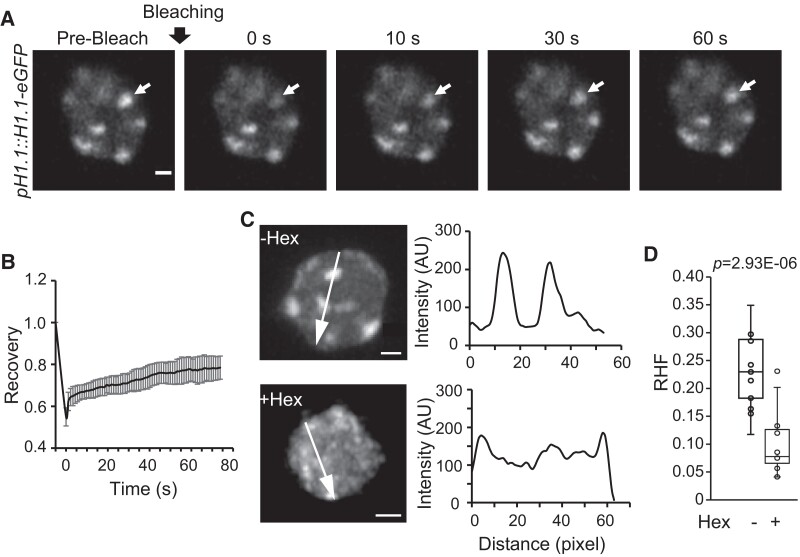
HF are gel-like condensates. **A)** FRAP on the nuclear body of H1.1-eGFP is indicated by the arrow. Scale bar, 1 *μ*m. **B)** Plot showing the recovery of H1.1-eGFP nuclear bodies after photobleaching. Data are presented as mean ± s.d. (*n* = 10). **C)** Airyscan images showing H1.1-eGFP nuclear bodies treated with or without Hex. The fluorescence intensities along the arrows are shown in arbitrary units (AU) on the right. **D)** Box plots depicting RHF computed with H1.1-eGFP intensity in nuclei treated with or without Hex. Each box encloses the middle 50% of the distribution, with the horizontal line marking the median and the vertical lines marking the minimum and maximum values that fall within 1.5 times the height of the box. *n* = 15 and 13 for the untreated and treated, respectively. Student's *t*-test *P* value is shown above.

We next investigated the properties of H1 foci in vivo. Our previous study showed that H1-eGFP (enhanced green fluorescent protein) driven by the native promoter colocalizes with HF (He et al. 2019). We took advantage of the transgenic lines for FRAP. In agreement with our in vitro experiments, after bleaching H1.1-eGFP and H1.2-eGFP signals are only mildly recovered (Fig. 4, A and B; Supplementary Fig. S6 and Movie S1), indicating gel-like properties of H1 condensates in vivo. Phase-separated condensates are often sensitive to 1,6-hexanediol (Hex), an aliphatic alcohol that specifically interferes with weak hydrophobic interactions (Strom et al. 2017; Long et al. 2021). Consistently, we observed that H1-eGFP foci in Arabidopsis root nuclei become dispersed upon Hex treatment (Fig. 4C). This was quantified by the relative heterochromatin fraction (RHF), which is defined as the relative fluorescence intensity of HF per total fluorescence of an entire nucleus. RHF is drastically reduced upon Hex treatment compared to the control (Fig. 4D). Together, these data demonstrate that H1 foci are phase-separated condensates with gel-like properties.

### C-terminal IDR mediates H1 phase separation

**Figure 5.**
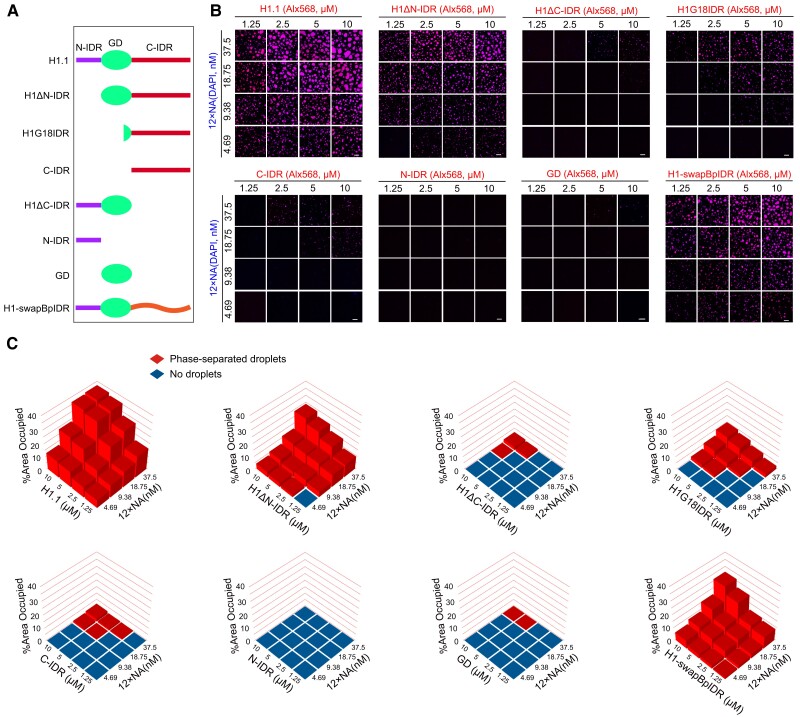
The C-IDR mediates H1 phase separation. **A)** Diagram of full-length, truncated, and chimeric H1.1 proteins used for in vitro phase separation assays. **B)** Phase diagrams of H1 variants as illustrated in (A) with 12 × nucleosome array (NA). The concentrations are indicated. H1 variants are labeled with Alx568 and DNA is stained with DAPI. The merged channel is shown. Scale bars, 10 *μ*m. **C)** Quantification of H1 variants’ phase separation with 12 × NA as shown in (B).

We sought to determine functional regions that mediate H1 phase separation. To this end, we purified recombinant H1.1 proteins with various truncations (Fig. 5A), and conducted their in vitro phase separation assays in the context of NA (Fig. 5B). Their phase separation capacity was quantified by the relative area occupied by phase-separated puncta (Fig. 5C). We observed that neither the N-terminal IDR (simplified as N-IDR) nor the globular domain (simplified as GD) can phase separate with NA (Fig. 5, B and C). The C-terminal IDR (simplified as C-IDR) alone also promotes hardly any NA puncta (Fig. 5, B and C), which is consistent with the knowledge that GD is required for the binding of H1 to linker DNA and nucleosomes (Allan et al. 1980). H1 lacking the C-IDR (abbreviated as H1ΔC-IDR), which contains both N-IDR and GD, forms negligible phase-separated puncta, showing that C-IDR is important for H1 phase separation capability (Fig. 5, B and C). In contrast, H1 with N-IDR deleted (abbreviated as H1ΔN-IDR) that includes both C-IDR and GD, promotes the formation of NA-H1 puncta, almost reaching the full phase separation capacity of intact H1 (Fig. 5, B and C). Strikingly, C-IDR with a very short stretch (18 out of 71 amino acids) of the GD (abbreviated as H1G18IDR; Fig. 5A) is sufficient to trigger substantial NA phase separation, evidencing the strong ability of C-IDR to stimulate chromatin phase separation (Fig. 5, B and C). Taken together, our results demonstrate that C-IDR greatly contributes to H1's phase separation ability, while the N-IDR contributes to a lesser extent, if at all.

### H1 phase separation drives HF formation

**Figure 6.**
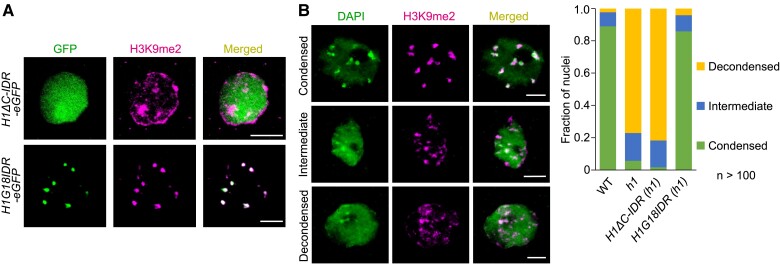
The C-IDR is required for HF formation. **A)** Immunostaining showing subnuclear localization of H1ΔC-IDR-eGFP and H1G18IDR-eGFP in *h1* mutant leaf nuclei. **B)** Examples of leaf nuclei with condensed, intermediate, or decondensed chromocentres, and their fractions in the indicated genotypes. H1ΔC-IDR and H1G18IDR indicate H1 with the C-IDR deleted and a truncated GD plus the C-IDR, respectively, as illustrated in Fig. 5A. Scale bars (A, B), 5 *μ*m.

To determine if H1 phase separation is required for HF formation, we generated *ProH1:H1ΔC-IDR-eGFP h1* lines by introducing a truncated H1.2 with the C-terminal IDR deleted into the *h1* mutant background. We observed that H1ΔC-IDR-eGFP is evenly spread throughout the nucleoplasm and forms no distinct foci (Fig. 6A), suggesting that the C-IDR is necessary for phase separation as well as preferential localization of H1 to heterochromatin. Furthermore, immunostaining with anti-H3K9me2 antibody shows that H1ΔC-IDR-eGFP failed to rescue the decondensed HF in *h1* (Fig. 6, A and B), suggesting that H1 phase separation is important for condensing HF. To further confirm this, we expressed H1G18IDR (C-IDR with a very short stretch of GD, as depicted in Fig. 5A), which demonstrated remarkable in vitro phase separation ability (Fig. 5, B and C), in the *h1* mutant background using H1 native promoter. Distinct H1G18IDR-eGFP foci, which colocalize with condensed HF, were observed in *ProH1:H1G18IDR-eGFP h1* leaf nuclei (Fig. 6A). These H1G18IDR-eGFP foci colocalize with condensed HF, which were largely abolished in the *h1* mutant but restored to WT level in *ProH1:H1G18IDR-eGFP h1* (Fig. 6B). The striking restoration achieved using the C-IDR and only a short stretch (18 out of 71 amino acids) of GD, highlights the importance of C-IDR for H1 function. The prominent differences between H1ΔC-IDR and H1G18IDR in HF formation are not caused by varying effects of these truncations on H1's DNA binding ability, as gel shift assays confirm that both variants effectively bind to DNA (Supplementary Fig. S7). Together, our results show that C-IDR-mediated H1 phase separation is required for HF formation.

### Regulation of nucleosome repeat length and DNA methylation requires the C-IDR of H1

**Figure 7.**
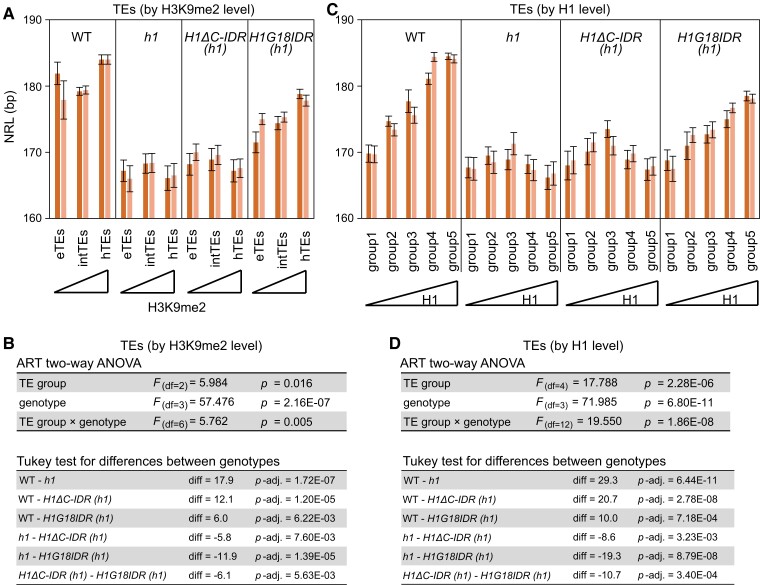
The C-IDR is required for H1 regulation of nucleosome repeat length. **A, C)** Nucleosome repeat length (NRL) for 2 biological replicates (two different batches of seedlings of the same genotypes) at TEs grouped by H3K9me2 (A) or H1 deposition (C) in WT. Error bars indicate standard error (SE). **B, D)** Aligned ranks transformation (ART) 2-way ANOVA followed by Tukey test for (A and C). df, degrees of freedom; diff, differences; eTEs, euchromatic TEs; intTEs, intermediate TEs; hTEs, heterochromatic TEs; H1ΔC-IDR and H1G18IDR indicates H1 with the C-IDR deleted and a truncated GD plus the C-IDR, respectively, as illustrated in Fig. 5A.

Next, we sought to explore how H1 phase separation contributes to known chromatin regulatory functions of H1. It is known that H1 increases nucleosome repeat length (NRL), particularly that of heterochromatic TEs where H1 is most abundant (Choi et al. 2020). We analyzed NRL by performing MNase-seq analyses with seedlings of WT, *h1*, *pH1::H1ΔC-IDR-eGFP h1*, and *pH1::H1G18IDR-eGFP h1*. We found that *H1ΔC-IDR* expression has little effect on the NRL in *h1*, and fails to rescue the preferential NRL increase over heterochromatic TEs (or TEs with most H1; Fig. 7, A to D), consistent with the lack of preferential deposition into heterochromatin of H1ΔC-IDR (Fig. 6A). In contrast, *H1G18IDR* expression in *h1* substantially increases NRL, with a clear preference for more heterochromatic TEs (or TEs with more H1; Fig. 7, A to D), which is in agreement with its colocalization with HF (Fig. 6A). Altogether, these results demonstrate that H1 C-IDR is essential for the NRL property in Arabidopsis. It is noteworthy that *H1G18IDR* did not fully rescue the NRL phenotype of *h1*, showing that the full-length H1 is required for full spacing between nucleosomes.

**Figure 8.**
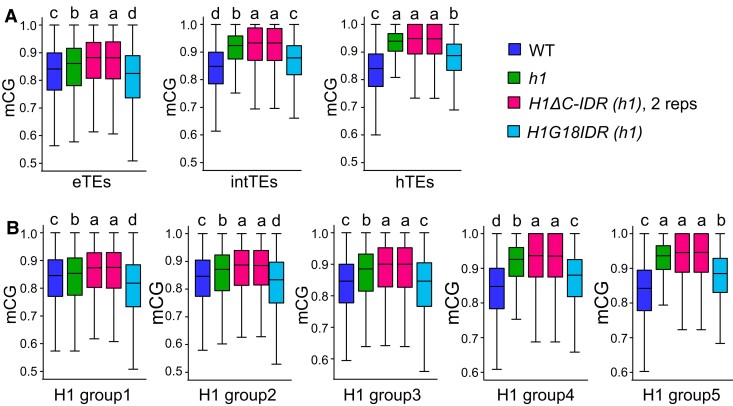
The C-IDR is required for H1 regulation of DNA methylation. **A, B)** Box plots show DNA methylation levels in 50-bp windows within different TE groups by H3K9me2 (A) or H1 deposition (B) in the indicated genotypes. *H1ΔC-IDR (h1)* has two replicates. Only windows with at least 10 informative sequenced cytosines and fractional methylation of at least 50% for CG, 30% for CHG, and 6% for CHH are included. Each box encloses the middle 50% of the distribution, with the horizontal line marking the median and vertical lines marking the 1.5× IQR or the minimum and maximum values that fall within 1.5× IQR. Values indicated by distinct letters are significantly different from each other (Welch ANOVA followed by unpaired *t* with Welch's correction, *P* < 0.01). eTEs, euchromatic TEs; intTEs, intermediate TEs; hTEs, heterochromatic TEs; H1ΔC-IDR and H1G18IDR indicates H1 with the C-IDR deleted and a truncated GD plus the C-IDR, respectively, as illustrated in Fig. 5A. H1 groups correspond to Fig. 7C.

To test the roles of C-IDR in mediating H1 regulation of DNA methylation, we performed bisulfite-sequencing with seedlings of the WT, *h1*, *ProH1:H1ΔC-IDR-eGFP h1*, and *ProH1:H1G18IDR-eGFP h1*. Consistent with previous reports (Zemach et al. 2013; Lyons and Zilberman 2017), our results show that CG methylation, particularly that of heterochromatic TEs, is increased in *h1* compared to in WT (Fig. 8A), as well as CHG and CHH methylation (H = A, T, C) of intermediate and heterochromatic TEs (Supplementary Fig. S8). Moreover, our data show that the expression of *H1G18IDR,* but not *H1ΔC-IDR*, partially rescues the DNA hypermethylation phenotype of the *h1* mutant (Fig. 8, A and B; Supplementary Fig. S8). The complete inability of *H1ΔC-IDR* to rescue *h1* DNA methylation, in contrast to the significant rescue by *H1G18IDR,* demonstrates that the effect of H1 on DNA methylation is reliant on the C-IDR.

### H1 phase separation likely represents an ancient mechanism for DNA condensation

Similar to Arabidopsis, loss of H1 also leads to HF decondensation in mouse embryonic stem cells and *Drosophila* (Lu et al. 2009; Cao et al. 2013). The C-terminal IDRs of chicken and calf H1 proteins have been shown to mediate DNA compaction in vitro via phase separation (Gibbs and Kriwacki 2018; Turner et al. 2018; Gibson et al. 2019). H1-like proteins also exist in eubacteria, in which DNA is stored in a nuclear-like membraneless structure known as the nucleoid (Kasinsky et al. 2001). These H1-like proteins lack the GD but resemble the C-terminal disordered regions of eukaryotic H1 proteins in being enriched with lysine, alanine, and proline (Kasinsky et al. 2001). Intriguingly, H1-like proteins in *Bordetella pertussis*, *Chlamydophila psittaci*, *Chlamydia trachomatis*, and *Coxiella burnetii* have been implicated in DNA condensation, which occurs at specific stages of their life cycles when these proteins are expressed (Kaul et al. 1992; Perara et al. 1992; Christiansen et al. 1993; Heinzen and Hackstadt 1996; Grieshaber et al. 2006). We examined the sequences of these H1-like proteins and found that they lack structured domains and are primarily composed of IDRs (Supplementary Fig. S9). Therefore, we speculate that H1-like proteins might have evolved the capability of phase separation to compact bacterial genomes. This capability may have been preserved and adapted for chromatin condensation in eukaryotes together with the emergence of the GD. To test this idea, we replaced the C-IDR of Arabidopsis H1.1 with the H1-like protein from *Bordetella pertussis* for in vitro phase separation assay (simplified as H1-swapBpIDR; Fig. 5A). Unlike H1ΔC-IDR, which failed to promote NA phase separation, H1-swapBpIDR nearly restored the full phase separation capability observed in intact H1 (Fig. 5, B and C), underscoring the phase separation property of the *Bordetella pertussis* H1-like protein. Based on existing knowledge and our results, we propose that phase separation of H1 or H1-like proteins may represent an ancient mechanism for DNA condensation.

## Discussion

In this study, we demonstrate that H1 condenses heterochromatin by increasing chromosomal interactions at pericentromeric regions (Fig. 1D). Loss of H1 disperses HF, significantly reducing such interactions while enhancing heterochromatin-euchromatin interactions (Fig. 1, B and C). This has profound effects on higher-order chromatin organization, including inducing aberrant interactions between the two arms of each chromosome across heterochromatin-enriched chromocenters (Fig. 1D). We further demonstrate that H1 aggregates heterochromatin into highly condensed foci via a phase separation mechanism that is largely dependent on the C-terminal IDR (Figs. 5 and 6). With the C-IDR deleted, H1 is no longer able to phase separate or aggregate HF (Figs. 5 and 6), indicating heterochromatin condensation requires the phase separation capability of H1. Furthermore, we demonstrate that C-IDR is crucial for H1 preferential localization in heterochromatin and its chromatin functions, including the regulation of nucleosome spacing and DNA methylation (Figs. 6 to 8). These observations suggest that C-IDR-endorsed H1 phase separation is the primary mechanism by which histone H1 promotes heterochromatin condensation or achieves its function in heterochromatin. This perspective highlights a crucial avenue for further exploration of H1’s functions.

Linker histone H1 is a central structural component of eukaryotic chromatin, facilitating higher-order chromatin folding. While the C- and N- termini of H1 proteins vary in sequence homology between variants and across species, the GD is fairly conserved (Allan et al. 1980; Kasinsky et al. 2001). It is well established that the GD is necessary and sufficient for nucleosome recognition via binding linker DNA (Allan et al. 1980). To dissect the functional relevance of H1 domains, we generated various versions of truncated H1. Unexpectedly, we found that although H1ΔC-IDR is unable to complement functions of H1 in condensing HF, increasing nucleosome repeat length, or regulating DNA methylation, H1G18IDR (which contains C-IDR and only a very short stretch of the GD) achieves a remarkable degree of complementation in all these aspects (Figs. 5 to 7 and 8). This not only demonstrates the importance of C-IDR for H1 functions but also suggests the relative insignificance of the GD, at least in Arabidopsis. Bacterial H1-like proteins lack the GD but have been implicated in DNA condensation (Kaul et al. 1992; Perara et al. 1992; Christiansen et al. 1993; Heinzen and Hackstadt 1996; Grieshaber et al. 2006), and our data suggest that they are intrinsically disordered and can phase separate (Fig. 5 and Supplementary Fig. S9). Based on these results, we propose that phase separation of bacterial H1-like proteins may represent an ancient mechanism for DNA/chromatin condensation. In eukaryotes, the adoption of a GD assists the IDR by achieving efficient linker DNA binding and H1 loading, however, the IDR is still indispensable for chromatin condensation and regulatory functions of H1. Consistent with the idea that the phase separation capability endowed by the IDR is critical for H1's chromatin functions, bacterial H1-like proteins and the C-IDR of animal and plant H1 proteins do not exhibit sequence conservation but do share common features, such as the absence of structured domains and a similar amino acid composition. Finally, it is also important to consider that the IDR may influence chromatin structure and function through other mechanisms beyond phase separation. For instance, the IDR could interact with various chromatin factors, including histone variants and chromatin remodelers (Fyodorov et al. 2018). These aspects merit further exploration in future studies.

## Materials and methods

### Plant materials and growth conditions

Arabidopsis (*Arabidopsis thaliana*) plants were grown under 16 h light/8 h dark in a growth chamber (20 °C, 80% humidity). All plants were of the Col-0 ecotype. *ProH1.1:H1.1-eGFP, ProH1.2:H1.2-eGFP*, and the *h1* (*h1.1 h1.2* double) mutant lines were described previously (She et al. 2013; Zemach et al. 2013)). *ProH1:H1ΔC-IDR-eGFP* (from position 1 to 124 in the amino acid sequence of H1.2) and *pH1::H1G18IDR-eGFP* (from position 111 to 274 in the amino acid sequence of H1.1) were constructed with InFusion system into the destination vector pCambia 1,300 and were introduced into the *h1* mutant background. We obtained two independent transgenic lines for each of the two constructs, which are *ProH1:H1ΔC-IDR-eGFP (h1)#1-2, ProH1:H1ΔC-IDR-eGFP (h1)#2-6, ProH1:H1G18IDR-eGFP (h1)#1-4,* and *ProH1:H1G18IDR-eGFP (h1)#2-1.* The T3 generation was used for the analyses on subcellular localization, HF formation, NRL, and DNA methylation. All primers used are presented in Supplementary Table S1.

### Fluorescence recovery after photobleaching

Roots of *ProH1.1:H1.1-eGFP* and *ProH1.2:H1.2-eGFP* seedlings were microscopically observed under the Zeiss 880 in Airyscan mode. Individual H1.1-eGFP and H1.2-eGFP bodies were bleached with a 488 nm laser pulse and recovery was recorded over time. Images were processed with ImageJ.

*In vitro* FRAP experiments were carried out with a NIKON A1 microscope equipped with a 100× oil immersion objective. Droplets were bleached with a 488- or 561-nm laser pulse (3 repeats, 70% intensity, dwell time 1 s). Recovery from photobleaching was recorded for the indicated time.

### Hex treatment

*ProH1.1:H1.1-eGFP* seedlings were treated with either 10% Hex (w/v) or the solvent MS medium for 10 min, and then roots were microscopically observed under the Zeiss 880 in Airyscan mode. Images were processed with ImageJ. Chromocentres were defined and the RHF was calculated with NucleusJ (Poulet et al. 2015).

### Protein purification from *E. coli*

H1.1, H1.2, and all forms of H1.1 variants, including truncated and chimeric H1, were cloned into a pET28a vector and expressed with a C-terminal 6xHis-tag in *Escherichia coli* strain BL21 (DE3) in the presence of 0.1 mM IPTG overnight at 16 °C. Cells were collected by centrifugation and resuspended in lysis buffer: 20 mM Tris–HCl, 500 mM NaCl, pH 8.0. The cells were then lysed by ultrasonication. After centrifugation at 18,000 × *g*, the supernatant was applied to a Chelating SFF(Ni) column, and target proteins were eluted with 250 mM Imidazole. The resultant proteins were further purified by cation-exchange chromatography using an SP HP column (GE Healthcare). The eluted peaks were applied to a Superdex 75 10/300GL (GE Healthcare) gel filtration column, then dialyzed and concentrated in a stock buffer: 20 mM HEPES, 150 mM NaCl, pH 8.0.

### Reconstitution of 12 × nucleosome array

Recombinant *Arabidopsis thaliana* core histones (H2A.1, H2B.2, H3, and H4) and DNA template of 12 tandem 177-bp repeats of the 601 sequence were cloned and purified as previously described (Li et al. 2010; Buttress et al. 2022). The histone octamers were reconstituted using the serial dialysis method of (Dyer et al. 2004), by combining equal molar amounts of individual histones and dialyzing them from unfolding buffer (20 mM HepesNa, pH 7.4, 6 M guanidine hydrochloride) into refolding buffer (2 M NaCl, 10 mM Tris–HCl, pH 8.0, 1 mM EDTA, 5 mM β-mercaptoethanol). Octamers were further purified through a Superdex 200 10/300 GL column (GE Healthcare). NA for phase separation assay was assembled using the salt dialysis method, as previously described (Wang et al. 2019). The reconstitution reaction of octamer and DNA templates was carried out at 4 °C overnight, from the Tris-EDTA-NaCl buffer (10 mM Tris–HCl, pH 8.0, 1 mM EDTA, 2 M NaCl) diluted by Tris-EDTA (TE) buffer (10 mM Tris–HCl, pH 8.0, 1 mM EDTA) to a lower concentration of NaCl buffer (10 mM Tris–HCl, pH 8.0, 1 mM EDTA, 0.6 M NaCl), followed by a final dialysis step in phase separation assay buffer (20 mM HEPES, pH 7.4, 100 mM NaCl) at 4 °C for 4 h.

### In vitro phase separation assay

Phase separation assays were performed in phase separation assay buffer (20 mM HEPES, pH 7.4, 100 mM NaCl). In vitro experiments were recorded on 384 low-binding multiwell 0.17 mm microscopy plates (In vitro Scientific) and sealed with optically clear adhesive film. Imaging was performed with a NIKON A1 microscope equipped with a 100× oil immersion objective. NIS-Elements AR Analysis and ImageJ were used to analyze these images.

### Immunofluorescence

*ProH1:H1ΔC-IDR-eGFP (h1)#1-2* and *ProH1:H1G18IDR-eGFP (h1)#1-4* were used for Immunofluorescence, which was performed exactly as described previously with Mouse anti-H3K9me2 (Abcam ab1220, 1:100) and Rabbit anti-GFP (Abcam ab290, 1:100) (He et al. 2019).

### Bisulfite-seq and analysis

DNA was extracted from 10-d-old seedlings of WT, *h1* mutant, *ProH1:H1ΔC-IDR-eGFP (h1)#1-2* and *ProH1:H1G18IDR-eGFP (h1)#1-4* using the CTAB method. Bisulfite-sequencing libraries were prepared with NEBNext Ultra II DNA Library Prep Kit and EZ DNA Methylation-Lightning Kit following the manufacturer's instructions. Illumina sequencing was performed on the NextSeq 500 (Illumina) with 75 bp single end at the John Innes Centre. Sequenced reads were mapped to the TAIR10 reference genome and cytosine methylation analysis was performed, as previously described (Hsieh et al. 2016; He et al. 2019).

### TE categories defined by H3K9me2 and H1 levels

TE categories (eTE, intTE, and hTE) defined by H3K9me2 levels were described previously (Hsieh et al. 2016). TE groups categorized by H1 levels (H1 groups) were defined with the cutoffs of H1 enrichment over TEs being −9.59 < Log_2_(ChAP/Input)<−0.33, −0.33 < Log_2_(ChAP/Input) < 0.25, 0.25 < Log_2_(ChAP/Input) < 0.88, 0.88 < Log_2_(ChAP/Input) < 1.8, Log_2_(ChAP/Input) > 1.8 for groups 1 to 5, respectively. Chromatin affinity purification (ChAP) sequencing data of H1 were generated, as previously described (Choi et al. 2020).

### MNase-seq and analysis

MNase-seq and analysis were performed as described (Lyons and Zilberman 2017; Choi et al. 2020). Briefly, 0.5 g of 10-d-old seedlings of WT, *h1* mutant, *ProH1:H1ΔC-IDR-eGFP (h1)#1-2*, and *ProH1:H1G18IDR-eGFP (h1)#1-4* were ground with mortar and pestle in liquid nitrogen and homogenized in 10 mL of nuclei isolation buffer (0.25 M sucrose, 15 mM PIPES pH 6.8, 5 mM MgCl_2_, 60 mM KCl, 15 mM NaCl, 1 mM CaCl_2_, 0.9% Triton X-100 (v/v), 1 mM PMSF, 1× proteinase inhibitors Cocktail) for 15 min on ice. Debris was filtered through 2 layers of miracloth to obtain a nuclei suspension, which was then cold centrifuged at 4,000 × *g* for 10 min at 4 °C. Nuclei were resuspended in 500 *µ*L of TM2 (50 mM Tris–HCl, 2 mM MgCl_2_, 0.25 M sucrose, 1 mM PMSF, 1×proteinase inhibitors Cocktail). After cold centrifugation at 4,000 × *g* for 5 min, nuclei were resuspended in 400 *µ*L of MNase digestion buffer (50 mM Tris–HCl pH 7.5, 5 mM CaCl_2_, 0.25 M sucrose, 1 mM PMSF, 1× proteinase inhibitors Cocktail) with 1,000 U/mL final concentration of MNase (New England Biolabs) and incubated at 37 °C with agitation (1,000 rpm on Thermomixer) for 10 min. Digestion was stopped by adding 40 *µ*L of 0.5 mM EDTA and 20 *µ*L of 10% SDS (w/v). Proteinase K and RNase A were added to remove proteins and RNAs before Phenol-chloroform extraction to obtain DNA. Libraries were prepared using Ovation Ultralow System V2 (Nugen) and sequenced on the NextSeq 500 (Illumina) with 2 × 38 bp paired ends at the John Innes Centre.

Sequencing reads were mapped to TAIR10 with Bowtie2. Only mononucleosomal fragments (between 130 and 200 bp) were retained. Nucleosome repeat length analysis was done as described previously (Choi et al. 2020). Briefly, NuMap was performed to compute dyad positions (Alharbi et al. 2014), which were then used to calculate NRL, phasogram, and linear regression fit in R (please refer to https://github.com/dblyons/MNase_seq for detailed scripts).

### IDR prediction

IDR was predicted with an online program, namely VLXT program in Pondr (http://www.pondr.com/). The protein information used for the prediction can be found in Supplementary Table S2.

### Hi-C library preparation and analysis

The Hi-C library construction and sequencing were conducted by Annoroad Gene Technology Co., Ltd (Beijing, China). Briefly, 10-d-old seedlings of WT and *h1* mutant were harvested and vacuum fixed in 20 mL of 2% formaldehyde (w/v) for 15 min at room temperature and quenched by adding 2.162 mL of 2.5 M glycine. Samples were rinsed three times with water. The nuclei were extracted as follows: samples were ground with a mortar and pestle in liquid nitrogen and resuspended with 25 mL of extraction buffer I (0.4 M sucrose, 10 mM Tris–HCl pH 8, 10 mM MgCl_2_, 5 mM β-mercaptoethanol, 0.1 mM PMSF, and 13 *μ*L protease inhibitors). The nuclear suspension was then filtered through miracloth (Calbiochem) and centrifuged at 4,000 rpm for 20 min at 4 °C. The supernatant was discarded, and the pellet was resuspended with 1 mL of extraction buffer II (0.25 M sucrose, 10 mM Tris–HCl, pH 8, 10 mM MgCl_2_, 1% Triton X-100 (v/v), 5 mM β-mercaptoethanol, 0.1 mM PMSF, and 13 *μ*L protease inhibitors). The mixture was centrifuged at 14,000 rpm for 10 min at 4 °C before resuspending the pellet with 300 *μ*L of extraction buffer III (1.7 m sucrose, 10 mM Tris–HCl, pH 8, 0.15% Triton X-100 (v/v), 2 mM MgCl_2_, 5 mM β-mercaptoethanol, 0.1 mM PMSF, and 1 *μ*L protease inhibitors). The mixture was loaded onto the top of an equal volume of clean extraction buffer III before centrifuging at 14,000 rpm for 10 min. The nuclei in the pellet were washed twice with 1× ice-cold CutSmart buffer and resuspended in 0.5 mL volume. SDS was applied to permeabilize nuclei at 65 °C for 10 min, then Triton X-100 was added to quench SDS. Thereafter, chromatin was digested with 400 units of MboI overnight at 37 °C on a rocking platform. MboI activity was denatured followed by DNA end repair with biotin-14-dCTP (deoxycytidine triphosphate) incorporation and blunt-end ligation. After decrosslinking with proteinase K at 65 °C, DNA was purified by phenol-chloroform extraction. Biotin-14-dCTP was removed from nonligated DNA fragment ends using T4 DNA polymerase. DNA was sheared to a size ranging from 200 to 600 bp by sonication. Thereafter, the fragments were end repaired and pulled down by streptavidin C1 magnetic beads to enrich those fragments containing biotin. Lastly, the fragment ends were A-tailed, ligated with sequencing adapters, and PCR amplified for 12 to 14 cycles. The libraries were cleaned up and sequenced on an Illumina HiSeq platform with a 2 × 150 bp read length.

For Hi-C data analysis, clean reads were mapped to the TAIR10 genome using HiC-Pro (version 2.11.1) pipeline (Servant et al. 2015). The bam files (bwt2merged.bam) generated by HiC-Pro, were used as input files for Fan-C (version 0.9.8) (Kruse et al. 2020). The module “fanc auto” was applied to generate contact matrixes (hic files) with various bin sizes. To obtain the matrix for comparative genotypes, we ran “fanc compare” with fold change and “fanc dump” sequentially. The output matrix was then visualized as a heatmap with ggplot2 in R. Compartmentalization of the genome was visualized in an enrichment profile plot. We divided 25 kb bins into percentiles using the associated eigenvector values, and then the average observed/expected values of contacts in each pair of percentile bins were calculated and plotted using the “fanc compartments" module. A compartmentalization switch assay between *h1* and WT was generated by comparing the ± characteristics for each 25-kb bin. The interactions between A/B compartments were further compared between the WT and h1 mutant by generating box plots.

### Accession numbers

Sequencing data generated in this study have been deposited into GEO (GSE176526).

## Supplementary Material

koae034_Supplementary_Data

## Supplementary data

The following materials are available in the online version of this article.

Supplementary Figure S1. Schematic diagram illustrating HF in WT and *h1* somatic nuclei. (Supports Figure 1.)

Supplementary Figure S2. Statistics of Hi-C reports. (Supports Figure 1.)

Supplementary Figure S3. Hi-C maps of WT and *h1* seedlings. (Supports Figure 1.)

Supplementary Figure S4. H1 does not phase separate on its own in vitro. (Supports Figure 2.)

Supplementary Figure S5. H1.1 and H1.2 mix in puncta in vitro. (Supports Figure 2.)

Supplementary Figure S6. FRAP on H1.2-eGFP. (Supports Figure 4.)

Supplementary Figure S7. Gel shift assays of truncated H1 with DNA. (Supports Figure 6.)

Supplementary Figure S8. Effects of H1 truncations on non-CG methylation of intermediate and heterochromatic TEs. (Supports Figure 8.)

Supplementary Figure S9. IDR prediction of bacterial H1-like proteins. (Supports Figure 5.)

Supplementary Table S1. Primers used in this study.

Supplementary Table S2. Protein sequences used for IDR prediction.

## Dive Curated Terms

The following phenotypic, genotypic, and functional terms are of significance to the work described in this paper:

MET1 Gramene: AT5G49160
MET1 Araport: AT5G49160

DAPI CHEBI: CHEBI:51231

HEPES CHEBI: CHEBI:46756

DDM1 Gramene: AT5G66750
DDM1 Araport: AT5G66750
