# Supplementary material for: Linker histone H1 drives heterochromatin condensation via phase separation in Arabidopsis
Source: Plant Cell. 2024 Feb 3;36(5):1829–43. doi: 10.1093/plcell/koae034 (PMC11062459; doi:10.1093/plcell/koae034)
Supplement: koae034_Supplementary_Data [file koae034_supplementary_data.zip › tpc.01020.2023-s06.pdf]

THE PLANT CELL

Phase separation of linker histone H1 is required for heterochromatin condensation in Arabidopsis

Xiaoqi Feng, Shengbo He, Yiming Yu, Liang Wang, Guohong Li, and Pilong Li

Review Timeline:

Submission Date:	21-Jan-2022
Editorial Decision:	03-Mar-2022
Resubmission Received:	01-Nov-2023
Accepted:	25-Nov-2023

Dr. Xiaoqi Feng
John Innes Centre
Norwich NR4 7UH
United Kingdom

Dear Xiaoqi / Dr. Feng:

Thank you for choosing to send your manuscript entitled "Linker histone H1 drives heterochromatin condensation via phase separation in Arabidopsis" for consideration at The Plant Cell. Your submission has been evaluated by members of the editorial board as well as expert reviewers in your field, and we regret to inform you that we are not able to recommend publication of this manuscript in its current form. We have had input from multiple scientists, and we have solicited post-review comments as well. Our present policy is to offer streamlined decisions and to not advise on the direction of the work by requesting extensive modifications or substantial additional experiments.

During the post-review consultation session, we also agreed that if you could address the major points raised by the reviewers by new experiments, we would welcome a resubmission. This may be treated as a new submission, but we would attempt to use at least some of the same reviewers. Nevertheless, reviewers will be asked to assess as a new manuscript (i.e. are the claims fully supported by the data and do the results presented move the field forward?), and not only whether previous reviewer comments have been addressed.

There was general enthusiasm about this study, but the reviewers universally agreed that in vitro phase separation assays of different H1 variants were required for a number of the figures. If data from these experiments can be added to this study, this will satisfy the major comments from the reviewers. If you decide to resubmit to The Plant Cell, please also place this study in better context with previous work on this topic published in animal systems, as noted by multiple reviewers. We're hopeful you'll be able to complete the requested revisions, but declining the study at this time provides you the opportunity to submit this study elsewhere if you chose not to.

It will be important to convince the editors and reviewers that the major claims made are fully justified by the data presented. This includes careful consideration and explanation of the various controls used in experiments, the extent and manner of replication, and the statistical analyses used. Sampling methods and the nature of "biological replicates" should be described precisely (i.e. different plants, parts of plants, pooled tissue, independent pools of tissue, sampled at different times, etc.), along with a clear description of and rationale for any statistical analyses conducted. The reader should know exactly what was sampled; what forms the basis of the calculation of any means and other statistical variables and parameters reported. This is also necessary to ensure that proper statistical analysis was conducted.

The Plant Cell now requires authors to complete and submit an author revisions checklist upon submission of a revised manuscript. The aim of the checklist is to aid authors in preparing a high quality manuscript, facilitate the review and assessment of revised manuscripts, and help to ensure that journal standards are maintained across the board. If your manuscript is accepted, the completed checklist will be published as supplemental material attached to the article online. Please download a copy of the checklist (a fillable PDF form) at this link, for submission with your revised manuscript: https://tpc.msubmit.net/html/Author_Revisions_Checklist.pdf.

Note also that supplemental materials should be restricted to large datasets and tables, presentation of replicates, and validation of reagents, methods, or genotypes. Any data that are used to support the main claims must be in the main manuscript. Supplemental figure legends must indicate what figure in the main manuscript is supported by the supplemental data presented.

We thank you for your interest in and support of The Plant Cell. We hope we will be able to render a more positive decision on future work.

On behalf of the editorial board,

Reviewing Editor, Board of Reviewing Editors
Bob Schmitz, Senior Editor
Blake Meyers, Editor-in-Chief

The Plant Cell

-- Reviewer comments:

Reviewer #1 (Comments for the Author):

In this manuscript He et al. seek to understand what redundant pathways are controlling chromatin compaction in Arabidopsis thaliana. The authors focus on the linker histone H1 due to its well-known role in compacting DNA. Further, it is not known completely how H1 facilitates chromatin compaction in plants. The authors show that the C-terminal intrinsically disordered region (IDR) of H1 is very important for chromatin compaction. Strikingly, the expression of the IDR of H1 along with a small portion of the histone globular domain is sufficient to rescue the decompaction phenotype. This data along with in vitro phase separation assays of H1 with 12x601 DNA and 12xnucleosome arrays bring the authors to conclude that H1 utilizes its IDR to compact DNA in a phase separation mediated process.

The clear demonstration that the IDR of H1 is important to compact plant heterochromatin is of interest. However, the authors should acknowledge in the discussion that the IDR could be mediating other processes as well, in addition to phase separation, such as modifying chromatin structure by interacting with DNA or nucleosomes or binding other chromatin proteins to help mediate the compaction. Therefore, there could be other possible interpretations of these results. Regardless, the phase-separation hypothesis is attractive, but the argument could be made stronger and clearer with the following changes and experiments:

1. The authors in vivo data showing that the IDR of H1 is important for the function of H1 is convincing, but to demonstrate that the IDR of H1 is truly necessary for phase separation the authors should replicate their in vitro phase separation assays using H1ΔIDR and the IDR only protein construct. The H1ΔIDR should not be able to phase separate in vitro.
2. The authors interpretation of in vitro H1 FRAP recovery is confusing. In the same paragraph the authors conclude both that H1 FRAP curves reach "a plateau after approximately 1 minute (Figure 3B), suggesting that they are liquid-like in behavior", and that the H1 FRAP curves lack a full recovery which "suggests that the initial liquid-like H1 puncta become less mobile and gel-like over time." These experiments seem to support a general gel-like state based on an initial recovery that is negligible compared to the overall signal before bleaching. Most likely the initial recovery is just an internal rearrangement of the puncta since the bleaching is not a total bleaching of the foci but instead is partial bleaching of the foci. Therefore, the data does not support the conclusion that this is "liquid-liquid" phase separation.
3. The authors write: "This is reminiscent of HF mediated by HP1a phase separation in Drosophila embryos (Larson et al., 2017; Strom et al., 2017)." This sentence only refers to Strom et al, while Larson et al is incorrectly cited. Moreover, the reference is not fully accurate because in this manuscript He et al do not show an initial liquid-like behavior that then becomes "gel-like".
4. The fact that the IDR with just a short stretch of the globular domain largely rescues the in vivo phenotypes but the delta IDR doesn't is surprising and very interesting, but essential controls for this experiment are lacking: the authors need to show that both the delta IDR and the "IDR only" are able to bind the linker DNA in vitro. Indeed, it could be that the H1ΔIDR doesn't even bind DNA, and in that case you would not expect it to be able to rescue any phenotype. In the discussion the authors say, "we demonstrate that IDR-endorsed phase separation ability of H1 is required for its preferential localization in heterochromatin and chromatin functions, including the regulation of nucleosome spacing and DNA methylation". Currently this conclusion cannot be made because the enrichment of the H1ΔIDR to the heterochromatin foci could be simply due to its ability to bind DNA (DNA density is higher in the heterochromatin foci).
5. It is not clear from the methods section what transgenic lines were used for the rescue experiments: which generation? How many independent lines? These details should be clarified in the methods.
6. Figures 6 and 7 contain interesting trends that seem to show expression of the H1 IDR/globular domain hybrid protein leads to trends of mCG and NRL that are similar to WT phenotypes. It's difficult to truly interpret these data without statistics to conclude that the differences are significant. It seems that the NRL of the H1 IDR/globular domain is different from both WT and the H1 deletion which would suggest that the full-length protein is necessary for full compaction, but this is difficult to conclude based on the information presented. Similar conclusion for mCG. At minimum it would be clearer if authors show the data points overlayed on top their graphs to be transparent.
7. It is not clear why the authors only show the group 2 and group 4 loci in supplementary figure 8. Please include all 5 groups in the figure.
8. The introduction of the bacterial H1 like proteins provides an interesting evolutionary perspective. The authors claim they "examined the phase separation properties of these H1-like proteins" by plotting disorder/order plots of the bacterial H1 protein amino acids but this is not the same as testing their phase separation properties. This is merely predicting whether there are structured regions of the protein. The sentence should be changed unless the authors have assays to support the possible phase separation capacity of the bacterial proteins otherwise the authors can only claim that the proteins are most likely disordered proteins without structured domains.

Reviewer #2 (Comments for the Author):

Phase separation and genome architecture are emerging research fields. Many chromatin structural proteins shape 3D genome architecture via the liquid-liquid phase separation (LLPS) mechanism. He et al. found histone H1 might promote chromatin interactions within the heterochromatin domain for condensation. They demonstrated that the C-terminal IDR of H1 protein could drive Heterochromatin Foci (HF) formation via DNA/NA-depend phase separation, playing an essential role in the H1 regulation of nucleosome repeat length and DNA methylation. The findings might facilitate our understanding of the function of H1-associated phase separation in promoting heterochromatin condensation. However, the authors should provide more deep

analysis and solid evidence, especially appropriate controls, to characterize the properties and functions of H1-associated LLPS.

Here are my specific comments:
1. As reported by Gibson et al. (2019, Cell), intrinsic chromatin properties such as linker DNA length or nucleosomal spacing and regulatory factors such as histone H1, acetylation, and multi-bromodomain proteins modulate chromatin LLPS, which could enable establishment and maintenance of distinct chromatin compartments. Since there are many factors that may regulate LLPS and chromatin condensation, the authors should provide more solid evidence to explain how H1 drives the heterochromatin condensation via phase separation. For the title, I think "Phase separation of the linker histone H1 is required for heterochromatin condensation in Arabidopsis" maybe better.
2. Lines 106−129, for Figure 2, the FRAP and puncta overlapping results, just as shown in Figure 3B−3C, are required.
3. For Figure 2 and Figure 3, the authors should add the results showing in vitro phase separation of H1ΔIDR and H1IDR to examine the function of IDR in driving H1 phase separation.
4. Lines 130−145, since H1.1 and H1.2 are two major H1 paralogs, and HF could rarely be observed in the H1.1-H1.2 double mutant, we recommend the authors to add the FRAP results of H1.2-eGFP plants.
5. In addition, the FRAP experiments showed quite low recovery rates, suggesting that the puncta might be gel-like. One may argue that the process should be referred to as phase transition (from liquid to solid), rather than liquid-liquid phase separation.
6. As mentioned above, we recommend the authors to add the IF results of wild type (H1.1-H1.2), h1.1 (h1.1-H1.2), h1.2 (H1.1-h1.2), and h1 (h1.1-h1.2) plants in Figure 5.
7. The authors used roots as experimental materials in the FRAP assays, while in the IF experiments, they took nuclei from leaves as input materials. Could the authors give some rationale or guidance for why one would be chosen over the other in different experiments?

Reviewer #3 (Comments for the Author):

The manuscript by He et al describes that biomolecular condensate formation of a histone protein is the fundamental driving force for heterochromatin formation in Arabidopsis. Phase separation is a timely subject and the mechanisms for nuclear body formation of heterochromatin has been a long-lasting question in the field of plant epigenetics. This study integrates these two research areas and answers to a relevant question in plant biology by presenting high-quality data of bioinformatics (HiC-seq, MNase-seq, and BS-seq) and biochemistry (phase separation assay). The manuscript is very well-written, and I have only few minor suggestions.

1. The authors started the Introduction very nicely emphasizing that the mutations in most known heterochromatin factors lead to only marginal decondensation of heterochromatin, while H1 plays a critical role for heterochromatin formation. Just to strengthen this notion, I think the authors' previous work published in Elife (2019) could be mentioned in the Introduction as an example of natural depletion of H1 in vegetative cell nuclei, which display strong heterochromatin decondensation. In addition, I would also suggest to include more introduction about liquid-liquid phase separation (LLPS) and the known cases of LLPS in chromatin factors in other systems (including Gibbs PNAS 2018 & Gibson Cell 2019).

2. This study includes many phase separation data, which are of overall high standard. However, I should point to some issues that require improvement and clarification:
- Phase separation of IDRs is only tested in vivo (Figure 4 & 5); in vitro phase separation data for H1 IDRs should be included in Figure 2 or 3. In addition, the authors seem to ignore the N terminal peaks of IDR prediction (presumably based on the in vivo data), but this could be tested in vitro without any huge difficulties. I'm also curious if the authors tested crowding agent such as PEG in their in vitro phase separation assay.
- I would like to encourage the authors to display the phase separation data in a more quantitative format, so that LLPS behavior can be compared more statistically. For example, droplet formation can be shown as average area of droplets or in a similar way that the authors performed in Figure 4D for relative heterochromatin fraction.
- FRAP data shown in this work only exhibit weak recovery and was interpreted simply as proteins being immobilized (line 128 & 137). However, the recovery rate can be dependent on the sizes of droplets and bleach spot (see doi: 10.1016/j.cell.2018.12.035). I would therefore encourage the authors to re-test FRAP with, for instance, smaller bleach spot. Besides, since it is becoming more common to provide quantitative data for phase separation, FRAP data can be also presented as t1/2 of recovery.
- With regards to IDR deletion, it is in fact difficult to directly conclude that IDR is required for H1 LLPS and function by truncating IDR. Particularly with IDRs, IDR-mediated LLPS is generally more acceptable when biased amino acids within IDRs are identified and loss of LLPS is demonstrated when these residues are mutated (refer to doi: 10.1101/gad.305227.117). Alternatively, the authors could test replacing the IDR with some other phase-separating domain with confirmed activity for LLPS to argue that LLPS is important for H1 function. Otherwise, the authors should avoid conclusive statements that H1 LLPS is important for its function (line 153-154, 161-162, 177-178) because IDR truncation does not necessarily demonstrate LLPS, and such statements should be rephrased in such a way that IDR is associated with H1 function.
- The authors used Widom 601 DNA and NA for in vitro phase separation assay, but I don't see any particular reasons for using it. I wonder if Arabidopsis DNA or chromatin was ever tested for in vitro phase separation experiments and if there are any

specific reasons for such experimental complexity.
- The authors stated that H1 LLPS is dependent on DNA but in Figure 2B the increase of DNA amount inhibits LLPS in lower concentration of H1. Can this be explained or clarified in the text?

3. The paragraph starting from line 193 could be moved to Discussion as it is rather speculative basing on mere prediction of phase separation domains. In addition, the sentence in line 212 should be rephrased to "examined the IDRs of these H1-like proteins" as LLPS can occur to proteins with no IDRs (DOI: 10.1016/j.cell.2020.01.011; DOI: 10.1016/j.cell.2020.07.037).

4. On more minor issues:
- Explain the arrowheads in Figure 3A.
- Provide number of nuclei examined in Figure 5B.
- Provide P values in Figure 6 and 7.
- Define the error bars in Figure 6 and 7.

We thank all reviewers for their constructive comments. We have performed new experiments, and analyses and revised the manuscript, accordingly. Below we respond in blue to each reviewer's specific comments, reproduced in italics.

Reviewer #1 (Comments for the Author):

In this manuscript He et al. seek to understand what redundant pathways are controlling chromatin compaction in Arabidopsis thaliana. The authors focus on the linker histone H1 due to its well-known role in compacting DNA. Further, it is not known completely how H1 facilitates chromatin compaction in plants. The authors show that the C-terminal intrinsically disordered region (IDR) of H1 is very important for chromatin compaction. Strikingly, the expression of the IDR of H1 along with a small portion of the histone globular domain is sufficient to rescue the decompaction phenotype. This data along with in vitro phase separation assays of H1 with 12x601 DNA and 12xnucleosome arrays bring the authors to conclude that H1 utilizes its IDR to compact DNA in a phase separation mediated process.

The clear demonstration that the IDR of H1 is important to compact plant heterochromatin is of interest. However, the authors should acknowledge in the discussion that the IDR could be mediating other processes as well, in addition to phase separation, such as modifying chromatin structure by interacting with DNA or nucleosomes or binding other chromatin proteins to help mediate the compaction. Therefore, there could be other possible interpretations of these results. Regardless, the phase-separation hypothesis is attractive, but the argument could be made stronger and clearer with the following changes and experiments:

We thank the reviewer for their positive comments and constructive suggestions. We agree and have now acknowledged in the discussion that the IDR might be mediating other processes than phase separation.

Lines 301-309:
"Consistent with the idea that the phase separation capability endowed by the IDR is critical for H1's chromatin functions, bacterial H1-like proteins and the C-IDR of animal and plant H1 proteins do not exhibit sequence conservation but do share common features, such as the absence of structured domains and a similar amino acid composition. Finally, it is also important to consider that the IDR may influence chromatin structure and function through other mechanisms beyond phase separation. For instance, the IDR could interact with various chromatin factors, including histone variants and chromatin remodelers (Fyodorov et al., 2018). These aspects merit further exploration in future studies."

1. The authors in vivo data showing that the IDR of H1 is important for the function of H1 is convincing, but to demonstrate that the IDR of H1 is truly necessary for phase separation the authors should replicate their in vitro phase separation assays using

H1ΔIDR and the IDR only protein construct. The H1ΔIDR should not be able to phase separate in vitro.

We thank the reviewer for raising this key point. As such, we have performed phase separation assays with various truncations of H1 in the context of nucleosome arrays (NA), including H1ΔIDR (renamed in our revised manuscript as H1ΔC-IDR for clarity; Fig. 5A) and the IDR-only protein (renamed in our revised manuscript as H1G18IDR for clarity; Fig. 5A). As expected by the reviewer, H1ΔC-IDR did not exhibit phase separation, while H1G18IDR did (Figs 5B and 5C), confirming C-IDR's essential role in facilitating phase separation. In addition, our new results show that the short N-terminal IDR (N-IDR) of H1 has a relatively minor role in mediating phase separation, if any.

We have presented these results in new Figs 5B and 5C, and have revised our results section as quoted below:

Lines 176-193:
"We sought to determine functional regions that mediate H1 phase separation. To this end, we purified recombinant H1.1 proteins with various truncations (Figure 5A), conducted their *in vitro* phase separation assays in the context of NA (Figure 5B). Their phase separation capacity was quantified by the relative area occupied by phase-separated puncta (Figure 5C). We observed that neither the N-terminal IDR (simplified as N-IDR) nor the globular domain (simplified as GD) can phase separate with NA (Figures 5B and 5C). The C-terminal IDR (simplified as C-IDR) alone also promotes hardly any NA puncta (Figures 5B and 5C), which is consistent with the knowledge that GD is required for the binding of H1 to linker DNA and nucleosomes (Allan et al., 1980). H1 lacking the C-IDR (abbreviated as H1ΔC-IDR), which contains both N-IDR and GD, forms negligible phase-separated puncta, showing that C-IDR is important for H1 phase separation capability (Figures 5B and 5C). In contrast, H1 with N-IDR deleted (abbreviated as H1ΔN-IDR) that includes both C-IDR and GD, promotes the formation of NA-H1 puncta, almost reaching the full phase separation capacity of intact H1 (Figures 5B and 5C). Strikingly, C-IDR with a very short stretch (18 out of 71 amino acids) of the GD, (abbreviated as H1G18IDR; Figure 5A), is sufficient to trigger substantial NA phase separation, evidencing the strong capability of C-IDR to stimulate chromatin phase separation (Figures 5B and 5C). Taken together, our results demonstrate that C-IDR greatly contributes to H1's phase separation capability, while the N-IDR contributes to a lesser extent, if at all."

2. The authors interpretation of in vitro H1 FRAP recovery is confusing. In the same paragraph the authors conclude both that H1 FRAP curves reach "a plateau after approximately 1 minute (Figure 3B), suggesting that they are liquid-like in behavior", and that the H1 FRAP curves lack a full recovery which "suggests that the initial liquid-like H1 puncta become less mobile and gel-like over time." These experiments seem to support a general gel-like state based on an initial recovery that is negligible compared to the overall signal before bleaching. Most likely the initial recovery is just an internal rearrangement of the puncta since the bleaching is not a total bleaching of the foci but

instead is partial bleaching of the foci. Therefore, the data does not support the conclusion that this is "liquid-liquid" phase separation.

We are grateful to the reviewer for pointing out this inconsistency. To address the physical properties of the condensates, we performed additional FRAP experiments (with partial, half and complete bleaching), as well as droplet fusion assays. The results indicate that, regardless of the bleaching method applied (partial, half, or total bleaching), H1 puncta consistently exhibit low levels of recovery with comparable recovery rates, indicating the gel-like nature of H1 puncta. Additionally, we observed that H1 droplets were slowly fusing, further supporting the gel-like property of the condensates. We have included these data in our new Figs 2D-F and 3B-D and revised our text, accordingly:

Lines 145-160:
"We next examined the physical properties of H1 condensates. H1 puncta exhibit a slow merging pattern upon contact, which does not achieve complete fusion even after 60 min (Figure 2D), suggesting a gel-like property. We subsequently performed fluorescence recovery after photobleaching (FRAP) experiments with individual puncta that were subjected to partial, half, or complete bleaching. Consistent with the gel-like property of H1 puncta, we observed consistently low levels of recovery (~28% to ~36%) with comparable half-recovery rates (t1/2=1.16 min), regardless of the extent of puncta bleaching (Figure 2E).

We further reconstituted nucleosome arrays (NA) using recombinant *Arabidopsis* core histones and observed similar puncta formation in the presence of H1 (Figure 3A). Moreover, FRAP experiments with partial, half, or complete bleaching of H1.1 puncta in the context of NA show similarly low levels (~23% to ~25.6%) of recovery and comparable half-recovery rates (t1/2=1.16 min for partial, 1.5 min for half, and 1.83 min for complete bleaching; Figures 3B and 3C) compared to in the context of DNA (Figures 2E and 2F), suggesting the gel-like nature of H1-mediated nucleosome phase separation. Consistently, NA-based H1.1 puncta show gradual and incomplete fusion over an extended duration (Figure 3D). Taken together, our data indicate that H1 undergoes gel-like phase separation in the presence of DNA/NA."

3. The authors write: "This is reminiscent of HF mediated by HP1a phase separation in Drosophila embryos (Larson et al., 2017; Strom et al., 2017)." This sentence only refers to Strom et al, while Larson et al is incorrectly cited. Moreover, the reference is not fully accurate because in this manuscript He et al do not show an initial liquid-like behavior that then becomes "gel-like".

We agree and have therefore removed the sentence.

4. The fact that the IDR with just a short stretch of the globular domain largely rescues the in vivo phenotypes but the delta IDR doesn't is surprising and very interesting, but essential controls for this experiment are lacking: the authors need to show that both the delta IDR and the "IDR only" are able to bind the linker DNA in vitro. Indeed, it could be that the H1ΔIDR doesn't even bind DNA, and in that case you would not expect

it to be able to rescue any phenotype. In the discussion the authors say, "we demonstrate that IDR-endorsed phase separation ability of H1 is required for its preferential localization in heterochromatin and chromatin functions, including the regulation of nucleosome spacing and DNA methylation". Currently this conclusion cannot be made because the enrichment of the H1ΔIDR to the heterochromatin foci could be simply due to its ability to bind DNA (DNA density is higher in the heterochromatin foci).

We thank the reviewer for their helpful suggestion. We have performed gel shift assays accordingly (new Supplemental Figure S7) and observed that both H1ΔIDR (renamed in our revised manuscript as H1ΔC-IDR for clarity; Fig. 5A) and the IDR-only protein (renamed in our revised manuscript as H1G18IDR for clarity; Fig. 5A) can bind DNA. We present this new result in the new Supplemental Figure S7 and describe it in the results section as follows:

Lines 210-213:
"The prominent differences between H1ΔC-IDR and H1G18IDR in HF formation are not caused by varying effects of these truncations on H1's DNA binding ability, as gel shift assays confirm that both variants effectively bind to DNA (Supplementary Figure S7)."

We acknowledge that the quoted statement was overly assertive and have revised it for enhanced accuracy in the discussion, accordingly:

Lines 273-280:
"With the C-IDR deleted, H1 is no longer able to phase separate or aggregate HF (Figures 5 and 6), indicating heterochromatin condensation requires the phase separation capability of H1. Furthermore, we demonstrate that C-IDR is crucial for H1 preferential localization in heterochromatin and its chromatin functions, including the regulation of nucleosome spacing and DNA methylation (Figures 6, 7, and 8). These observations suggest that C-IDR-endorsed H1 phase separation is the primary mechanism by which histone H1 promotes heterochromatin condensation or achieves its function in heterochromatin."

5. It is not clear from the methods section what transgenic lines were used for the rescue experiments: which generation? How many independent lines? These details should be clarified in the methods.

We apologize for the oversight in omitting this information in our previous manuscript version. We have now included this information in the methods section:

Lines 319–323:
"We obtained two independent transgenic lines for each of the two constructs, which are *pH1::H1ΔC-IDR-eGFP (h1)#1-2*, *pH1::H1ΔC-IDR-eGFP (h1)#2-6*, *pH1::H1G18IDR-eGFP (h1)#1-4*, and *pH1::H1G18IDR-eGFP (h1)#2-1*. The T3 generation was used for the analyses on subcellular localization, HF formation, NRL, and DNA methylation."

6. Figures 6 and 7 contain interesting trends that seem to show expression of the H1 IDR/globular domain hybrid protein leads to trends of mCG and NRL that are similar to WT phenotypes. It's difficult to truly interpret these data without statistics to conclude that the differences are significant. It seems that the NRL of the H1 IDR/globular domain is different from both WT and the H1 deletion which would suggest that the full-length protein is necessary for full compaction, but this is difficult to conclude based on the information presented. Similar conclusion for mCG. At minimum it would be clearer if authors show the data points overlayed on top their graphs to be transparent.

We appreciate this helpful suggestion. As such, we have performed the statistical analysis and incorporated the statistics into the two figures (Figs 7 and 8 in our revised manuscript). Additionally, we have attempted to add data points to the graphs as suggested. However, as illustrated in the representative graph below, the abundance of data points makes it challenging to discern individual points and blurs the boxes. Therefore, we have chosen not to include the data points directly on the graphs.

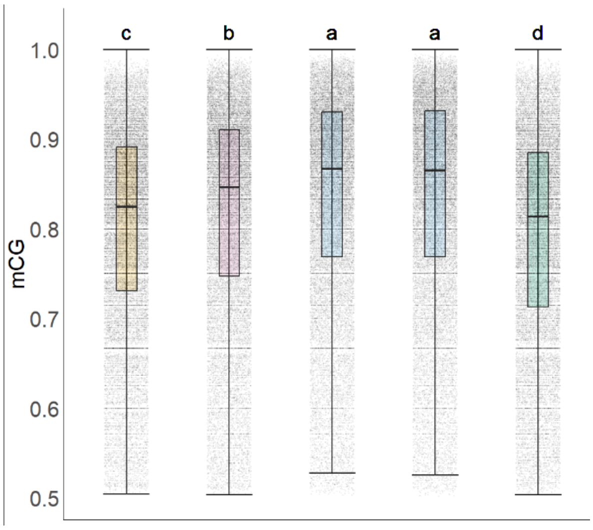

eTEs (Figure 8A left panel)

7. It is not clear why the authors only show the group 2 and group 4 loci in supplementary figure 8. Please include all 5 groups in the figure.

In our previous manuscript version, we showed Groups 1, 3, and 5 in Figure 7, and Groups 2 and 4 in Supplementary Figure S8. To avoid confusion, we have deleted Supplemental Figure S8 and added Groups 2 and 4 to the main figure (new Figure 8B).

8. The introduction of the bacterial H1 like proteins provides an interesting evolutionary perspective. The authors claim they "examined the phase separation properties of these H1-like proteins" by plotting disorder/order plots of the bacterial H1 protein amino acids but this is not the same as testing their phase separation properties. This is merely predicting whether there are structured regions of the protein. The sentence should be changed unless the authors have assays to support the possible

phase separation capacity of the bacterial proteins otherwise the authors can only claim that the proteins are most likely disordered proteins without structured domains.

We agree with the reviewer. We have tried to express these bacterial H1-like proteins for phase separation assays but we did not manage to obtain soluble proteins. However, these H1-like proteins lack structured domains, have high Pondr scores, and resemble the C-IDR of eukaryotic H1 proteins in being highly enriched with lysine, alanine and proline. Therefore, we took an alternative approach to test their phase separation capacity, by replacing the C-IDR of *Arabidopsis* H1.1 with the full-length bacterial H1-like protein sequence (H1-swapBpIDR; Fig. 5A). Strikingly, we observed almost fully restored phase separation capacity in the chimeric protein compared to the native H1.1 (while no phase separation capacity in H1ΔC-IDR), demonstrating the phase separation capability of the bacterial H1-like protein. We have included this new result in the new Figs 5B and 5C and described it in our revised manuscript as follows:

Lines 257-264:
"To test this idea, we replaced the C-IDR of *Arabidopsis* H1.1 with the H1-like protein from *Bordetella pertussis* for *in vitro* phase separation assay (simplified as H1-swapBpIDR; Figure 5A). Unlike H1ΔC-IDR, which failed to promote NA phase separation, H1-swapBpIDR nearly restored the full phase separation capability observed in intact H1 (Figures 5B and 5C), underscoring the phase separation property of the *Bordetella pertussis* H1-like protein. Based on existing knowledge and our results, we propose that phase separation of H1 or H1-like proteins may represent an ancient mechanism for DNA condensation."

Reviewer #2 (Comments for the Author):

Phase separation and genome architecture are emerging research fields. Many chromatin structural proteins shape 3D genome architecture via the liquid-liquid phase separation (LLPS) mechanism. He et al. found histone H1 might promote chromatin interactions within the heterochromatin domain for condensation. They demonstrated that the C-terminal IDR of H1 protein could drive Heterochromatin Foci (HF) formation via DNA/NA-depend phase separation, playing an essential role in the H1 regulation of nucleosome repeat length and DNA methylation. The findings might facilitate our understanding of the function of H1-associated phase separation in promoting heterochromatin condensation. However, the authors should provide more deep analysis and solid evidence, especially appropriate controls, to characterize the properties and functions of H1-associated LLPS.

Here are my specific comments:
1. As reported by Gibson et al. (2019, Cell), intrinsic chromatin properties such as linker DNA length or nucleosomal spacing and regulatory factors such as histone H1, acetylation, and multi-bromodomain proteins modulate chromatin LLPS, which could enable establishment and maintenance of distinct chromatin compartments. Since there

are many factors that may regulate LLPS and chromatin condensation, the authors should provide more solid evidence to explain how H1 drives the heterochromatin condensation via phase separation. For the title, I think "Phase separation of the linker histone H1 is required for heterochromatin condensation in Arabidopsis" maybe better.

We thank the reviewer for their constructive comments. We have performed a series of new experiments that have significantly improved the manuscript, accordingly. Most importantly: 1) we have performed nucleosome array phase separation assays with six different truncated versions of H1 proteins, demonstrating that C-terminal IDR (C-IDR) is essential for the phase separation capacity of H1 (Fig. 5); 2) we have conducted phase separation assays using a chimeric H1, in which the C-IDR is replaced with a bacterial H1-like protein (HLP) sequence, and have shown that HLP has phase separation capability, which may be responsible for DNA condensation in bacteria (Fig. 5); 3) we have performed a substantial array of FRAP and droplet fusion assays to determine that H1 condensates have a gel-like property (Figs 2 and 3). These new results, together with the strong *in planta* data that H1 C-IDR is indispensable for heterochromatin condensation and the regulation of nucleosome repeat length (NRL) and DNA methylation, clearly demonstrate that the phase separation of linker histone H1 is required for heterochromatin condensation (we have revised the manuscript title as suggested by the reviewer). However, we do agree that there are other possibilities and have added the following description to our revised discussion:

Lines 301-309:
"Consistent with the idea that the phase separation capability endowed by the IDR is critical for H1's chromatin functions, bacterial H1-like proteins and the C-IDR of animal and plant H1 proteins do not exhibit sequence conservation but do share common features, such as the absence of structured domains and a similar amino acid composition. Finally, it is also important to consider that the IDR may influence chromatin structure and function through other mechanisms beyond phase separation. For instance, the IDR could interact with various chromatin factors, including histone variants and chromatin remodelers (Fyodorov et al., 2018). These aspects merit further exploration in future studies."

2. Lines 106−129, for Figure 2, the FRAP and puncta overlapping results, just as shown in Figure 3B−3C, are required.

We thank the reviewer for this constructive suggestion. Accordingly, we have added FRAP and puncta overlapping results for Figure 2 (as new Figs 2D-2F).

3. For Figure 2 and Figure 3, the authors should add the results showing in vitro phase separation of H1ΔIDR and H1IDR to examine the function of IDR in driving H1 phase separation.

We thank the reviewer for the helpful suggestion. We have accordingly performed phase separation assays with various truncations of H1 in the context of nucleosome arrays (NA), including H1ΔIDR (renamed in our revised manuscript as H1ΔC-IDR for clarity; Fig. 5A) and H1IDR (renamed in our revised manuscript as H1G18IDR for

clarity; Fig. 5A). As expected by the reviewer, H1ΔC-IDR did not exhibit phase separation, while H1G18IDR did (Figs 5B and 5C), confirming C-IDR's essential role in facilitating phase separation. In addition, our new results show that the short N-terminal IDR (N-IDR) of H1 has a relatively minor role in mediating phase separation, if any.

We have presented these results in new Figs 5B and 5C, and have revised our results section as quoted below:

Lines 176-193:
"We sought to determine functional regions that mediate H1 phase separation. To this end, we purified recombinant H1.1 proteins with various truncations (Figure 5A), conducted their *in vitro* phase separation assays in the context of NA (Figure 5B). Their phase separation capacity was quantified by the relative area occupied by phase-separated puncta (Figure 5C). We observed that neither the N-terminal IDR (simplified as N-IDR) nor the globular domain (simplified as GD) can phase separate with NA (Figures 5B and 5C). The C-terminal IDR (simplified as C-IDR) alone also promotes hardly any NA puncta (Figures 5B and 5C), which is consistent with the knowledge that GD is required for the binding of H1 to linker DNA and nucleosomes (Allan et al., 1980). H1 lacking the C-IDR (abbreviated as H1ΔC-IDR), which contains both N-IDR and GD, forms negligible phase-separated puncta, showing that C-IDR is important for H1 phase separation capability (Figures 5B and 5C). In contrast, H1 with N-IDR deleted (abbreviated as H1ΔN-IDR) that includes both C-IDR and GD, promotes the formation of NA-H1 puncta, almost reaching the full phase separation capacity of intact H1 (Figures 5B and 5C). Strikingly, C-IDR with a very short stretch (18 out of 71 amino acids) of the GD, (abbreviated as H1G18IDR; Figure 5A), is sufficient to trigger substantial NA phase separation, evidencing the strong capability of C-IDR to stimulate chromatin phase separation (Figures 5B and 5C). Taken together, our results demonstrate that C-IDR greatly contributes to H1's phase separation capability, while the N-IDR contributes to a lesser extent, if at all."

4. Lines 130−145, since H1.1 and H1.2 are two major H1 paralogs, and HF could rarely be observed in the H1.1-H1.2 double mutant, we recommend the authors to add the FRAP results of H1.2-eGFP plants.

We have performed FRAP for H1.2-eGFP and added the results to Supplemental Figure S6, accordingly.

5. In addition, the FRAP experiments showed quite low recovery rates, suggesting that the puncta might be gel-like. One may argue that the process should be referred to as phase transition (from liquid to solid), rather than liquid-liquid phase separation.

We thank the reviewer for raising this important point. To address the physical properties of the condensates, we performed additional FRAP experiments (with partial, half and complete bleaching), as well as droplet fusion assays. The results indicate that, regardless of the bleaching method applied (partial, half, or total bleaching), H1 puncta consistently exhibit low levels of recovery with comparable recovery rates, indicating

the gel-like nature of H1 puncta as the reviewer suggested. Additionally, we observed that H1 droplets were slowly fusing, further supporting the gel-like property of the condensates. We have included these data in our new Figs 2D-F and 3B-D and revised our text, accordingly. We have also removed all mention of "liquid-liquid phase separation" in the manuscript and just referred to it as "phase separation."

Lines 145-160:
"We next examined the physical properties of H1 condensates. H1 puncta exhibit a slow merging pattern upon contact, which does not achieve complete fusion even after 60 min (Figure 2D), suggesting a gel-like property. We subsequently performed fluorescence recovery after photobleaching (FRAP) experiments with individual puncta that were subjected to partial, half, or complete bleaching. Consistent with the gel-like property of H1 puncta, we observed consistently low levels of recovery (~28% to ~36%) with comparable half-recovery rates (t1/2=1.16 min), regardless of the extent of puncta bleaching (Figure 2E).

We further reconstituted nucleosome arrays (NA) using recombinant *Arabidopsis* core histones and observed similar puncta formation in the presence of H1 (Figure 3A). Moreover, FRAP experiments with partial, half, or complete bleaching of H1.1 puncta in the context of NA show similarly low levels (~23% to ~25.6%) of recovery and comparable half-recovery rates (t1/2=1.16 min for partial, 1.5 min for half, and 1.83 min for complete bleaching; Figures 3B and 3C) compared to in the context of DNA (Figures 2E and 2F), suggesting the gel-like nature of H1-mediated nucleosome phase separation. Consistently, NA-based H1.1 puncta show gradual and incomplete fusion over an extended duration (Figure 3D). Taken together, our data indicate that H1 undergoes gel-like phase separation in the presence of DNA/NA."

6. As mentioned above, we recommend the authors to add the IF results of wild type (H1.1-H1.2), h1.1 (h1.1-H1.2), h1.2 (H1.1-h1.2), and h1 (h1.1-h1.2) plants in Figure 5.

We thank the reviewer for this suggestion. Firstly, H1.1 and H1.2 share high sequence similarities, resembling expression patterns, and comparable genomic localization, and are regarded in the field as closely related homologs with overlapping functions (PMID: 26351307, PMID: 23540698, PMID: 31135340, PMID: 31391082). We apologize for having not made this aspect clearer in the previous manuscript but we have revised the manuscript to highlight this.

Lines 95-98:
"In *Arabidopsis*, there are three H1 paralogs: H1.1 and H1.2, two major paralogs with a high degree of sequence similarity and overlapping functions in chromatin compaction, which are ubiquitously expressed in most tissues, and H1.3, which is specifically activated in response to stress conditions (Rutowicz et al., 2015)."

Lines 136-144:
"In *Arabidopsis*, H1.1 and H1.2 exhibit overlapping roles in maintaining heterochromatin condensation and are deposited to similar genomic regions (He et al.,

2019; Rutowicz et al., 2019; Choi et al., 2020). When H1.1 is added to pre-existing H1.2/DNA condensates or vice versa, all three of which are tagged with different fluorophores, condensates become homogenously triple-labelled over a prolonged period (Figure 2C and Supplemental Figure S5). This observation shows that the two H1 isoforms can coexist and intermingle within the same phase-separated condensates. This result aligns with the overlapping functions of H1.1 and H1.2 *in planta* and their substantial sequence similarities and shared expression patterns (Zemach et al., 2013; Rutowicz et al., 2015; Rutowicz et al., 2019)."

Secondly, two complications would interfere with the proposed experiments: 1) H1.2 protein level is enhanced in the *h1.1* single mutant compared to WT (PMID: 23540698), suggesting compensatory effects between the two paralogs, which would hinder their functional analysis; 2) The available *h1.2* mutant, that is commonly used in the community, has a T-DNA insertion in its promoter. This allele is not null and still produces a significant amount of H1.2 protein (PMID: 23540698), and therefore cannot be used to study H1.2 function. By contrast, when combined with the *h1.1* null mutant, the *h1.2* allele in the *h1.1 h1.2* double mutant produces little H1.2 protein (despite normal transcription) for an unknown reason, as reported in PMID: 23540698. For this reason, the double mutant has always been used to study H1 functions (PMID: 23540698, PMID: 31135340).

7. The authors used roots as experimental materials in the FRAP assays, while in the IF experiments, they took nuclei from leaves as input materials. Could the authors give some rationale or guidance for why one would be chosen over the other in different experiments?

We thank the reviewer for raising this question. We used roots for FRAP assays because roots are more amenable to live imaging than leaves. IF was performed with leaves as established previously for testing H1's role in heterochromatin foci formation (PMID: 31391082, PMID: 31732458, PMID: 31135340). In our experiments, we did not observe any difference in the localization of H1 to heterochromatin between roots and leaves.

Reviewer #3 (Comments for the Author):

The manuscript by He et al describes that biomolecular condensate formation of a histone protein is the fundamental driving force for heterochromatin formation in Arabidopsis. Phase separation is a timely subject and the mechanisms for nuclear body formation of heterochromatin has been a long-lasting question in the field of plant epigenetics. This study integrates these two research areas and answers to a relevant question in plant biology by presenting high-quality data of bioinformatics (HiC-seq, MNase-seq, and BS-seq) and biochemistry (phase separation assay). The manuscript is very well-written, and I have only few minor suggestions.

We thank the reviewer for their positive evaluation.

1. The authors started the Introduction very nicely emphasizing that the mutations in most known heterochromatin factors lead to only marginal decondensation of heterochromatin, while H1 plays a critical role for heterochromatin formation. Just to strengthen this notion, I think the authors' previous work published in Elife (2019) could be mentioned in the Introduction as an example of natural depletion of H1 in vegetative cell nuclei, which display strong heterochromatin decondensation. In addition, I would also suggest to include more introduction about liquid-liquid phase separation (LLPS) and the known cases of LLPS in chromatin factors in other systems (including Gibbs PNAS 2018 & Gibson Cell 2019).

We thank the reviewer for their helpful suggestions. We have added a paragraph in the introduction describing the relevant literature, accordingly (incl. He 2019 eLife, Gibbs PNAS 2018, and Gibson Cell 2019).

Lines 49-52:
"Depletion of H1 occurs naturally in the Arabidopsis vegetative cell, and this depletion leads to the complete dispersal of HF and the derepression of transposable elements (He et al., 2019; He and Feng, 2022)."

Lines 70-84:
"Phase separation has been shown to promote chromatin compartmentalization in both animals and plants (Wang et al., 2023). For instance, histone H2B.8 triggers a distinctive form of euchromatin condensation through phase separation in *Arabidopsis* sperm (Buttress et al., 2022). H3K9me3/2 readers, such as Heterochromatin Protein 1α (HP1α) and HP1β in human and HP1a in fruit fly, are suggested to condense heterochromatin via promoting phase separation (Larson et al., 2017; Strom et al., 2017; Wang et al., 2019; Wang et al., 2023). The corresponding reader in *Arabidopsis*, ADCP1, has also been shown to undergo phase separation (Zhao et al., 2019). However, as mentioned above, *adcp1* mutations have a limited effect on HF (Zhao et al., 2019), significantly less than *h1* mutations. Chicken and calf H1 proteins have been shown to promote phase separation of DNA and reconstituted nucleosome arrays through the C-terminal intrinsically disordered regions (C-IDRs) *in vitro*, respectively (Gibbs and Kriwacki, 2018; Turner et al., 2018; Gibson et al., 2019). However, if and how C-IDRs contribute to the phase separation capability of H1 *in vivo* has not been tested. Furthermore, it remains unknown if phase separation is a primary mechanism by which histone H1 promotes HF condensation or achieves its function in heterochromatin."

2. This study includes many phase separation data, which are of overall high standard. However, I should point to some issues that require improvement and clarification:
- Phase separation of IDRs is only tested in vivo (Figure 4 & 5); in vitro phase separation data for H1 IDRs should be included in Figure 2 or 3. In addition, the authors seem to ignore the N terminal peaks of IDR prediction (presumably based on the in vivo data), but this could be tested in vitro without any huge difficulties. I'm also curious if the authors tested crowding agent such as PEG in their in vitro phase separation assay.

We thank the reviewer for these constructive comments. We have performed phase separation assays accordingly, with various truncations of H1 in the context of nucleosome arrays (NA), including truncations explicitly testing the capacity of the N-terminal IDR (N-IDR), as suggested by the reviewer (Fig. 5A). The results demonstrate that C-IDR is critical for the phase separation ability of H1, while the N-IDR has a relatively minor role, if any. We did not try a crowding agent in our system as H1 exhibits strong phase separation capacity in the absence of any crowding agent.

We have presented these results in new Figs 5B and 5C, and have revised our results section as quoted below:

Lines 176-193:
"We sought to determine functional regions that mediate H1 phase separation. To this end, we purified recombinant H1.1 proteins with various truncations (Figure 5A), conducted their *in vitro* phase separation assays in the context of NA (Figure 5B). Their phase separation capacity was quantified by the relative area occupied by phase-separated puncta (Figure 5C). We observed that neither the N-terminal IDR (simplified as N-IDR) nor the globular domain (simplified as GD) can phase separate with NA (Figures 5B and 5C). The C-terminal IDR (simplified as C-IDR) alone also promotes hardly any NA puncta (Figures 5B and 5C), which is consistent with the knowledge that GD is required for the binding of H1 to linker DNA and nucleosomes (Allan et al., 1980). H1 lacking the C-IDR (abbreviated as H1ΔC-IDR), which contains both N-IDR and GD, forms negligible phase-separated puncta, showing that C-IDR is important for H1 phase separation capability (Figures 5B and 5C). In contrast, H1 with N-IDR deleted (abbreviated as H1ΔN-IDR) that includes both C-IDR and GD, promotes the formation of NA-H1 puncta, almost reaching the full phase separation capacity of intact H1 (Figures 5B and 5C). Strikingly, C-IDR with a very short stretch (18 out of 71 amino acids) of the GD, (abbreviated as H1G18IDR; Figure 5A), is sufficient to trigger substantial NA phase separation, evidencing the strong capability of C-IDR to stimulate chromatin phase separation (Figures 5B and 5C). Taken together, our results demonstrate that C-IDR greatly contributes to H1's phase separation capability, while the N-IDR contributes to a lesser extent, if at all."

- I would like to encourage the authors to display the phase separation data in a more quantitative format, so that LLPS behavior can be compared more statistically. For example, droplet formation can be shown as average area of droplets or in a similar way that the authors performed in Figure 4D for relative heterochromatin fraction.

We thank the reviewer for their excellent suggestion. We have included the quantifications in Fig. 5C and believe that this has strongly improved our phase separation data and their interpretation.

- FRAP data shown in this work only exhibit weak recovery and was interpreted simply as proteins being immobilized (line 128 & 137). However, the recovery rate can be dependent on the sizes of droplets and bleach spot (see doi: 10.1016/j.cell.2018.12.035). I would therefore encourage the authors to re-test FRAP with, for instance, smaller

bleach spot. Besides, since it is becoming more common to provide quantitative data for phase separation, FRAP data can be also presented as t1/2 of recovery.

We thank the reviewer for their constructive suggestions. We have performed new FRAP experiments accordingly, with partial, half, and complete bleaching (Figs 2E and 3B) and calculated recovery rates and half recovery times (t1/2; Figs 2F and 3C). We observed that H1 puncta have similar low levels of recovery with comparable t1/2, indicating H1 puncta are gel-like. We have also performed new droplet fusion assays, the results of which also support the gel-like properties of the condensates (Figs 2D and 3D). We have included these data in our new Figures 2 and 3 and revised our text, accordingly:

Lines 145-160:
"We next examined the physical properties of H1 condensates. H1 puncta exhibit a slow merging pattern upon contact, which does not achieve complete fusion even after 60 min (Figure 2D), suggesting a gel-like property. We subsequently performed fluorescence recovery after photobleaching (FRAP) experiments with individual puncta that were subjected to partial, half, or complete bleaching. Consistent with the gel-like property of H1 puncta, we observed consistently low levels of recovery (~28% to ~36%) with comparable half-recovery rates (t1/2=1.16 min), regardless of the extent of puncta bleaching (Figure 2E).

We further reconstituted nucleosome arrays (NA) using recombinant *Arabidopsis* core histones and observed similar puncta formation in the presence of H1 (Figure 3A). Moreover, FRAP experiments with partial, half, or complete bleaching of H1.1 puncta in the context of NA show similarly low levels (~23% to ~25.6%) of recovery and comparable half-recovery rates (t1/2=1.16 min for partial, 1.5 min for half, and 1.83 min for complete bleaching; Figures 3B and 3C) compared to in the context of DNA (Figures 2E and 2F), suggesting the gel-like nature of H1-mediated nucleosome phase separation. Consistently, NA-based H1.1 puncta show gradual and incomplete fusion over an extended duration (Figure 3D). Taken together, our data indicate that H1 undergoes gel-like phase separation in the presence of DNA/NA."

- With regards to IDR deletion, it is in fact difficult to directly conclude that IDR is required for H1 LLPS and function by truncating IDR. Particularly with IDRs, IDR-mediated LLPS is generally more acceptable when biased amino acids within IDRs are identified and loss of LLPS is demonstrated when these residues are mutated (refer to doi: 10.1101/gad.305227.117). Alternatively, the authors could test replacing the IDR with some other phase-separating domain with confirmed activity for LLPS to argue that LLPS is important for H1 function. Otherwise, the authors should avoid conclusive statements that H1 LLPS is important for its function (line 153-154, 161-162, 177-178) because IDR truncation does not necessarily demonstrate LLPS, and such statements should be rephrased in such a way that IDR is associated with H1 function.

We thank the reviewer for raising this point. Accordingly, we swapped the C-IDR of H1 by the full-length *Bordetella pertussis* H1-like protein (abbreviated as H1-swapBpIDR; Figure 5A), which does not have a globular domain and is mostly

intrinsically disordered (Supplementary Figure S9). We found that, unlike H1ΔC-IDR which fails to induce NA phase separation, H1-swapBpIDR promotes efficient phase separation, almost to the level of native H1 (Figs 5B and 5C). This demonstrates C-IDR mainly contributes to chromatin condensation via a phase separation mechanism.

Despite this, we do agree with the reviewer that other IDR-mediated processes might be involved in mediating H1 functions. Hence we have rephrased the sentences as proposed by the reviewer (at new Lines 201-202, 213-214; the phrase at original Lines 177-178 is removed), and we have included the following sentences in the discussion section:

Lines 301-309:
"Consistent with the idea that the phase separation capability endowed by the IDR is critical for H1's chromatin functions, bacterial H1-like proteins and the C-IDR of animal and plant H1 proteins do not exhibit sequence conservation but do share common features, such as the absence of structured domains and a similar amino acid composition. Finally, it is also important to consider that the IDR may influence chromatin structure and function through other mechanisms beyond phase separation. For instance, the IDR could interact with various chromatin factors, including histone variants and chromatin remodelers (Fyodorov et al., 2018). These aspects merit further exploration in future studies."

- The authors used Widom 601 DNA and NA for in vitro phase separation assay, but I don't see any particular reasons for using it. I wonder if Arabidopsis DNA or chromatin was ever tested for in vitro phase separation experiments and if there are any specific reasons for such experimental complexity.

We have not tried *Arabidopsis* DNA or chromatin for *in vitro* phase separation assays. Widom 601 DNA has a high affinity for histone octamers. Multiple copies of Widom 601 DNA have strong nucleosome positioning ability and therefore have been commonly used for *in vitro* nucleosome array assembly and phase separation assays (PMID: 31543265, PMID: 30425322, PMID: 36323776). Endogenous DNA or chromatin is more heterogenous than Widom 601 DNA or reconstituted NA in terms of fragment length, base composition and DNA sequence, and would therefore introduce complexity into *in vitro* assays.

- The authors stated that H1 LLPS is dependent on DNA but in Figure 2B the increase of DNA amount inhibits LLPS in lower concentration of H1. Can this be explained or clarified in the text?

We thank the reviewer for raising this question. Without DNA added, H1 cannot phase separate even at very high concentrations (e.g., 15 μM, Supplemental Figure S4). By contrast, low concentrations (< 2 μM) of H1 undergo phase separation in the presence of DNA (Fig. 2B), indicating that H1 phase separation is dependent on DNA. Our data show that phase separation can only occur at appropriate ratios of H1 to DNA, with either excess H1 or excess DNA interfering with phase separation. We have explained this in our revised manuscript as follows:

Lines 130-135:
"To test this, we performed an *in vitro* phase separation assay and found that H1.1 and H1.2 cannot phase separate on their own even under high concentrations (eg. 15 µM; Supplemental Figure S4). In contrast, the addition of DNA induces puncta formation, with as low as 0.25 µM of H1 forming condensates with DNA (Figure 2B). Phase separation of H1-DNA condensates occurs in a concentration-dependent manner (Figure 2B), indicating specific ratios of H1 to DNA are required."

3. The paragraph starting from line 193 could be moved to Discussion as it is rather speculative basing on mere prediction of phase separation domains. In addition, the sentence in line 212 should be rephrased to "examined the IDRs of these H1-like proteins" as LLPS can occur to proteins with no IDRs (DOI: 10.1016/j.cell.2020.01.011; DOI: 10.1016/j.cell.2020.07.037).

We have performed an *in vitro* phase separation assay with a bacterial H1-like protein (HLP) replacing the C-IDR of *Arabidopsis* H1 and observed that the HLP restores the phase separation capacity. This result shows that HLP has phase separation capability, which supports our hypothesis that HLPs likely represent an ancient mechanism of DNA condensation. We have revised our results section significantly to include this new data (Figs 5A-C) and to better explain our hypothesis and experiments (Lines 252-264). We have also revised the sentence the reviewer referred to, accordingly (now reads: "examined the sequences of these H1-like proteins"; Lines 252-253).

Lines 252-264:
"We examined the sequences of these H1-like proteins and found that they lack structured domains and are primarily composed of intrinsically disordered regions (Supplemental Figure S9). Therefore, we speculate that H1-like proteins might have evolved the capability of phase separation to compact bacterial genomes. This capability may have been preserved and adapted for chromatin condensation in eukaryotes together with the emergence of the globular domain (GD). To test this idea, we replaced the C-IDR of *Arabidopsis* H1.1 with the H1-like protein from *Bordetella pertussis* for *in vitro* phase separation assay (simplified as H1-swapBpIDR; Figure 5A). Unlike H1ΔC-IDR, which failed to promote NA phase separation, H1-swapBpIDR nearly restored the full phase separation capability observed in intact H1 (Figures 5B and 5C), underscoring the phase separation property of the *Bordetella pertussis* H1-like protein. Based on existing knowledge and our results, we propose that phase separation of H1 or H1-like proteins may represent an ancient mechanism for DNA condensation."

4. On more minor issues:
- Explain the arrowheads in Figure 3A.
- Provide number of nuclei examined in Figure 5B.
- Provide P values in Figure 6 and 7.
- Define the error bars in Figure 6 and 7.

All of these issues have been revised.

- Arrowheads in Fig. 3A have been removed as droplet fusion assays have been added

instead in Fig. 3D.

- The number of nuclei have been added to the revised Fig. 6B.

- P values are provided in the revised Figs. 7 and 8.

- Error bars are defined in the revised Figs. 7 and 8.

Dear Dr. Xiaoqi Feng:

We are pleased to inform you that your paper entitled "Phase separation of linker histone H1 is required for heterochromatin condensation in Arabidopsis" has been accepted for publication in The Plant Cell, pending a final editorial review by a science editor. At this stage, your manuscript will be evaluated by a science editor with respect to its presentation of scientific content, compliance with journal policies, and presentation for a broad readership. The Plant Cell has contracted with Plant Editors (planteditors.com) to provide this service to our authors, and you will soon receive additional information on it this process.

A note from the Editor-in-Chief: We are trying to make a concerted effort to change green/red comparisons to green/magenta to make our figures understandable to those with color vision deficiencies. We noticed that a small number of your figures utilize red/green contrasts, so as you prepare the final version of the figures, please check the figures for red/green color use. Magenta is a good substitute for red, and a light blue substitutes for green even if the data are labeled as "RFP" or "mCherry" - readers will understand that colors can be changed, and indeed, it can be adjusted relatively quickly by using the "hue" setting in Photoshop or similar software. For example, the following figures may need to be corrected: Figure 1A, Figure 6; you might want to check over the Supplemental Figures for similar comparisons. Please note that color changes do not need to be highlighted or tracked in the revised manuscript, but could be noted in the cover letter or response document.

Please note that each author needs to link their ORCID identifier to their account in the system before your manuscript can be published. If any authors do not have an ORCID linked to their account, they will receive a message with a link to complete this task. Please ensure that ALL of your coauthors have completed this task as soon as possible.

ASPB offers an OPEN option that allows authors to have their online articles available for free to all users immediately upon publication. For more information about the ASPB OPEN option, refer to the Final Submission Checklist Form.

The Plant Cell and The Arabidopsis Information Resource (TAIR) are collaborating to collect functional annotation data about Arabidopsis genes from authors. This includes information about the gene's molecular function (e.g., kinase activity, ATP synthetase activity), the biological process/es it is involved in (e.g., endosperm development, threonine biosynthesis), its subcellular location (e.g., nucleus, ER), anatomical or developmental expression pattern (e.g., leaf, ovule, flower stage 10, seedling stage), or its partner in a protein-protein interaction (e.g., AT1G01010 interacts with AT1G01020).

If your paper contains results falling into one or more of these categories for Arabidopsis genes, we request that you now submit these data for inclusion in TAIR by filling in the form provided at the following URL: https://www.arabidopsis.org/doc/submit/functional_annotation/123. If you need further clarification on what types of data can be submitted please contact curator@arabidopsis.org.

Finally, we encourage your submission of artwork for the journal cover. Monthly issues will have an online cover image and selected covers will be used for posters, other promotional items, and "wallpaper" for mobile devices. For more information, refer to Cover Submission in the Instructions for Authors https://tpc.msubmit.net/cgi-bin/main.plex?form_type=display_auth_instructions.

We look forward to seeing your paper published.

On behalf of the editorial

Robert Schmitz, Senior Editor
Blake Meyers, Editor-in-Chief

The Plant Cell

=====
IMPORTANT REMINDER: PEER REVIEW REPORTS
=====

If you opted to publish a peer review report along with your article during the original submission process, it will be prepared by the editorial staff and publicly posted with your manuscript, inside the zip file that contains any other supplemental material. As a reminder, the peer review report is a public record of all comments from editors and reviewers, as well as your prior responses, as you received them in the decision letters for each draft of your manuscript. If you agreed to publish this report and have changed your mind, or are not sure if you selected this option, please contact the editorial office as soon as possible before signing the license agreement from our publisher.

=====

REVIEWER COMMENTS
=====

Reviewer #1 (Comments for the Author):

The authors have done an excellent job of addressing my concerns.

Reviewer #3 (Comments for the Author):

The authors have addressed all my concerns, and I do not have any further issues to raise.

----FOR ASPB OFFICE USE ONLY (DO NOT EDIT)----
MSID: 36788
Corresponding Author: Prof. Dr. Feng
