# Supplementary material for: Linker histone H1 drives heterochromatin condensation via phase separation in Arabidopsis
Source: Plant Cell. 2024 Feb 3;36(5):1829–43. doi: 10.1093/plcell/koae034 (PMC11062459; doi:10.1093/plcell/koae034)
Supplement: koae034_Supplementary_Data [file koae034_supplementary_data.zip › TPC2023RA01020D_Supplemental_figures_and_tables.pdf]

Supplemental Data. He et al. (2024). H1 condenses heterochromatin by phase separation. Plant Cell.

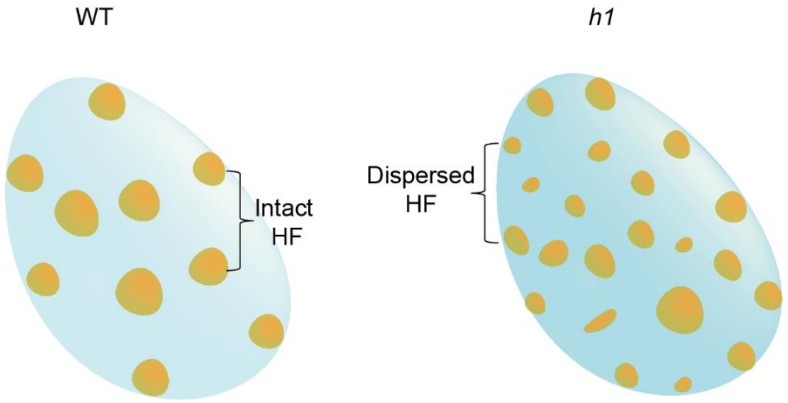

Supplemental Figure S1. Schematic diagram illustrating heterochromatin foci (HF) in WT and *h1* somatic nuclei. (Supports Figure 1.)
HF in the WT nucleus are intact, whereas those in the *h1* mutant nucleus are dispersed into more numerous and smaller HF. The darker background in the *h1* nucleus illustrates dispersed heterochromatin that fail to form foci.

Supplemental Data. He et al. (2024). H1 condenses heterochromatin by phase separation. Plant Cell.

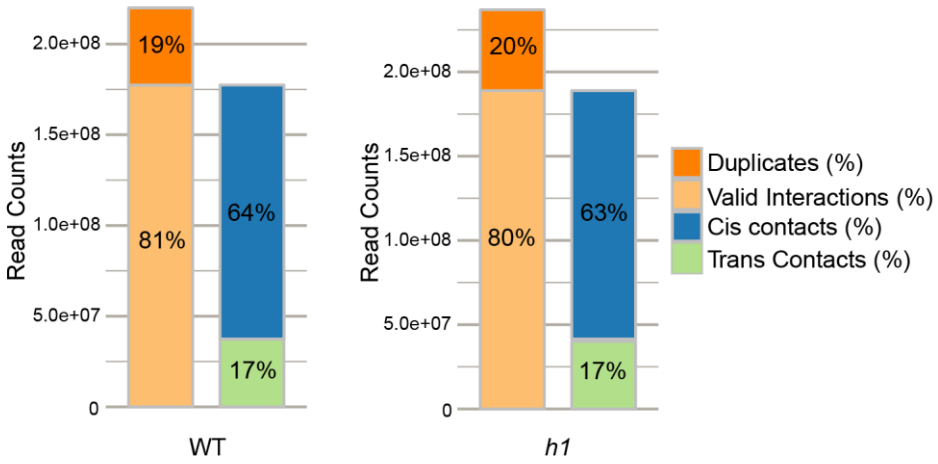

Supplemental Figure S2. Statistics of Hi-C reports. (Supports Figure 1.)
Histograms showing the percentages of duplicates, valid interactions, and *cis* (intra-chromosomal) as well as *trans* (inter-chromosome) interactions out of the total.

Supplemental Data. He et al. (2024). H1 condenses heterochromatin by phase separation. Plant Cell.

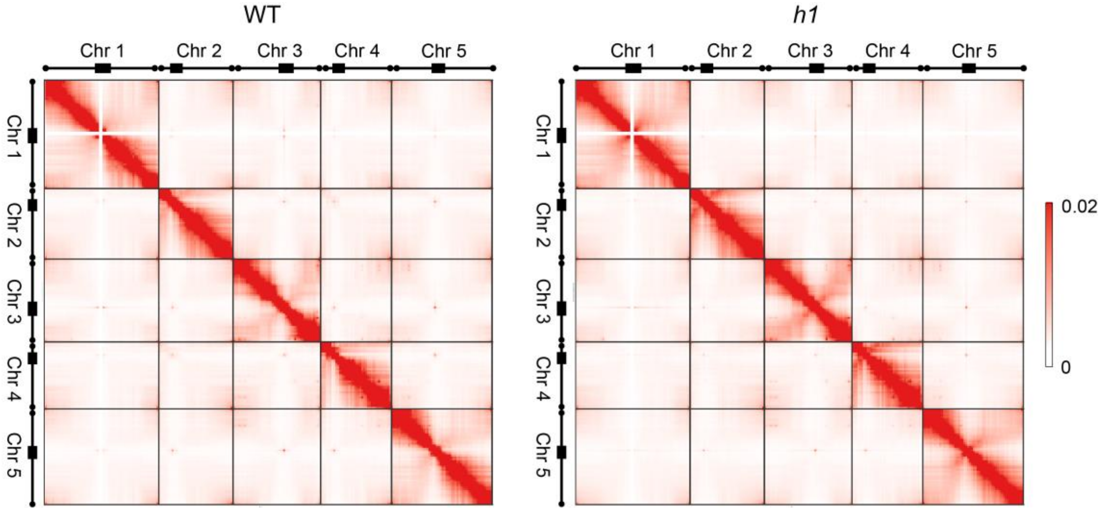

Supplemental Figure S3. Hi-C maps of wild-type and *h1* seedlings. (Supports Figure 1.)
Hi-C maps of the indicated genotypes showing normalized genome-wide interactions. Color bar denotes normalized interaction frequency.

Supplemental Data. He et al. (2024). H1 condenses heterochromatin by phase separation. Plant Cell.

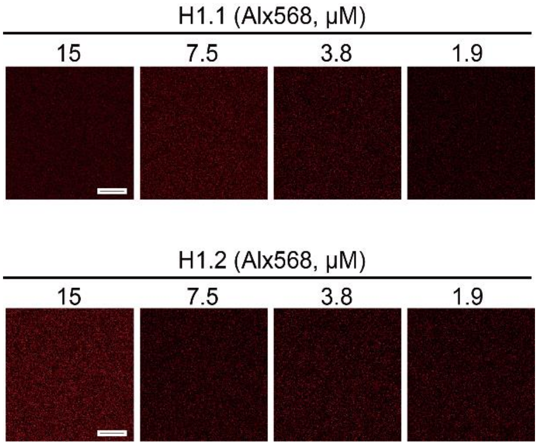

Supplemental Figure S4. H1 does not phase separate on its own *in vitro*. (Supports Figure 2.) Microscopic images of different concentrations of H1.1 and H1.2 labelled with Alx568. Scale bars, 5 μm.

Supplemental Data. He et al. (2024). H1 condenses heterochromatin by phase separation. Plant Cell.

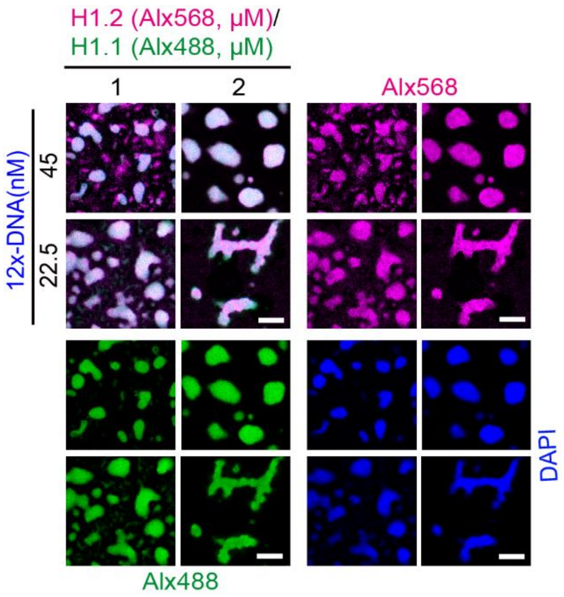

Supplemental Figure S5. H1.1 and H1.2 mix in puncta *in vitro*. (Supports Figure 2.)
Phase diagram showing H1.1 and H1.2 mix *in vitro* when H1.2 was added to pre-existing H1.1 puncta and incubated overnight. H1.1 and H1.2 were labelled with Alx488 and Alx568, respectively. DNA was stained with DAPI. Scale bars, 5 µm.

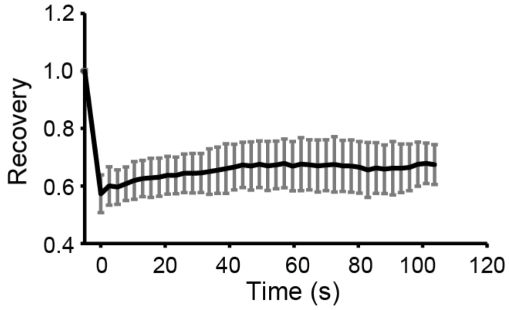

Supplemental Figure S6. FRAP on H1.2-eGFP. (Supports Figure 4.)

Plot showing the recovery of H1.2-eGFP nuclear bodies after photobleaching. Data are presented as mean ± s.d. (*n*=10).

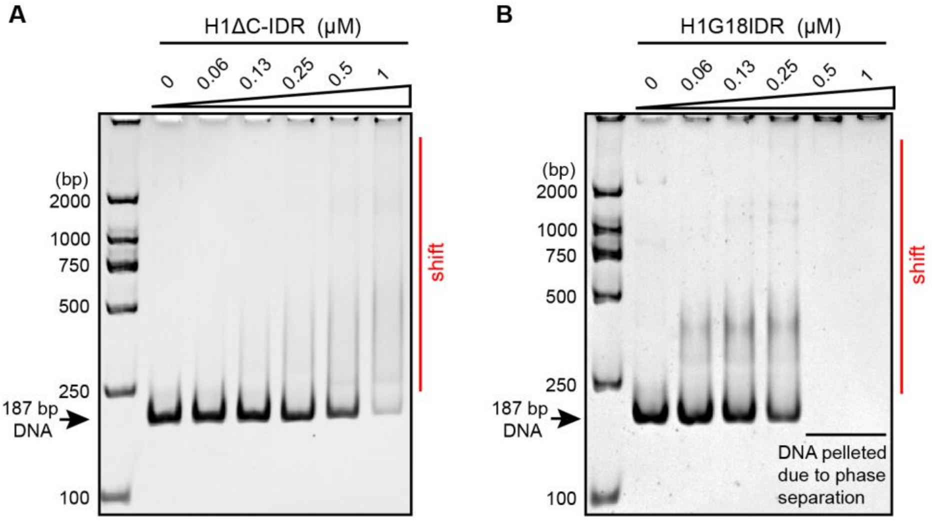

Supplemental Figure S7. Gel shift assays of truncated H1 with DNA. (Supports Figure 6.)

(A, B) Native agarose gel electrophoresis showing the shift of DNA by truncated H1 proteins under the indicated concentrations. Free DNA and protein-bound DNA shifts are indicated. Phase separation of H1G18IDR with DNA under high concentrations (0.5 and 1 μM) causes DNA-protein condensates not to enter the gel, hence missing DNA. H1ΔC-IDR and H1G18IDR indicate H1 with the C-IDR deleted and a truncated globular domain plus the C-IDR, respectively, as illustrated in Figure 5A.

Supplemental Data. He et al. (2024). H1 condenses heterochromatin by phase separation. Plant Cell.

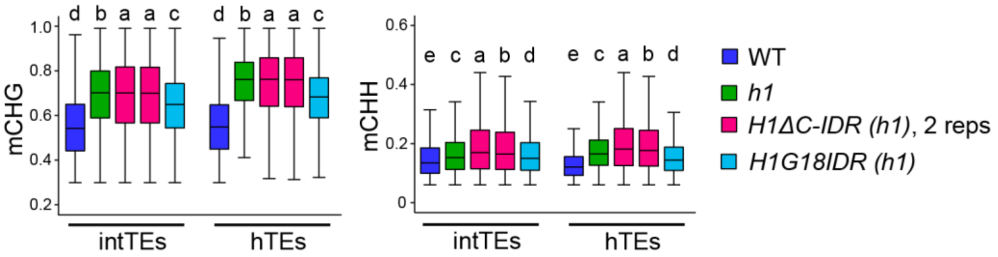

Supplemental Figure S8. Effects of H1 truncations on non-CG methylation of intermediate and heterochromatic TEs. (Supports Figure 8.)

Box plots show DNA methylation levels in 50-bp windows within different TE groups in the indicated genotypes. *H1ΔC-IDR (h1)* has two replicates. Only windows with at least 10 informative sequenced cytosines and fractional methylation of at least 30% for CHG and 6% for CHH are included. Each box encloses the middle 50% of the distribution, with the horizontal line marking the median and vertical lines marking the 1.5× interquartile range (IQR) or the minimum and maximum values that fall within 1.5× IQR. Values indicated by distinct letters are significantly different (Welch ANOVA followed by unpaired *t* with Welch's correction, $P<0.01$). intTEs, intermediate TEs; hTEs, heterochromatic TEs; H1ΔC-IDR and H1G18IDR indicates H1 with the C-IDR deleted and a truncated globular domain plus the C-IDR, respectively, as illustrated in Figure 5A.

Supplemental Data. He et al. (2024). H1 condenses heterochromatin by phase separation. Plant Cell.

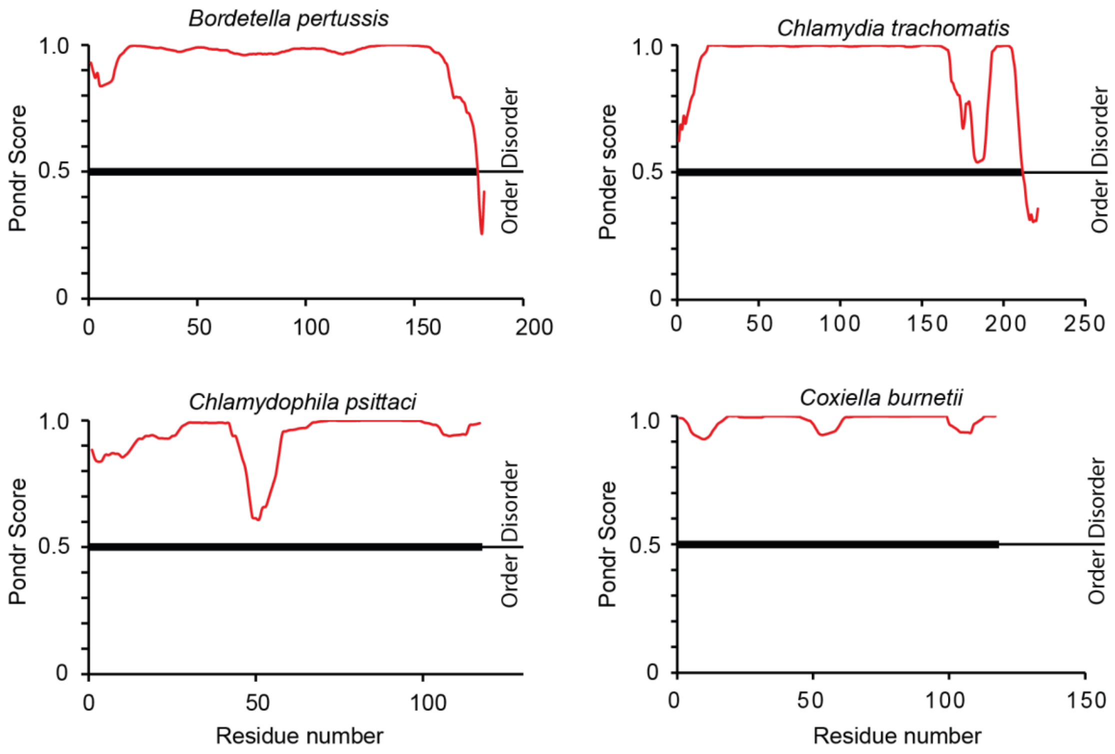

Supplemental Figure S9. IDR prediction of bacterial H1-like proteins. (Supports Figure 5.) Predictor of natural disordered regions (PONDR) scores for H1-like proteins from the indicated species. A score > 0.5 is considered disordered.

Supplemental Data. He et al. (2024). H1 condenses heterochromatin by phase separation. Plant Cell.

Supplemental Table S1. Primers used in this study.

Primer	Construct	Sequence (5' to 3')
PSH381	*pCambia1300-eGFP*	GAGCTCGGTACCCGGGGATCCGTG AGCAAGGGCGAGGAGC
PSH382	*pCambia1300-eGFP*	GGGGAAATTCTTACTTGTACAGCTC GTCCATGCC
PSH383	*pCambia1300-eGFP*	GTACAAGTAAGAATTTCCCCGATCG TTCAAAC
PSH384	*pCambia1300-eGFP*	ACGACGGCCAGTGCCAAGCTCCGA TCTAGTAACATAGATGACACC
PSH391	*pCambia1300-ProH1:H1ΔC-IDR-eGFP*	TATGACCATGATTACGAATTCTTCGT AAATGGTAGATGGAAAACA
PSH417	*pCambia1300-ProH1:H1ΔC-IDR-eGFP*	CTATAGACATCTTCTTCTCTCTCAGA AACTGATCT
PSH418	*pCambia1300-ProH1:H1ΔC-IDR-eGFP*	AGAGAAGAAGATGTCTATAGAGGAA GAAAACGTTC
PSH419	*pCambia1300-ProH1:H1ΔC-IDR-eGFP*	CTCGCCCTTGCTCACGGATCCAGCT TTGACTTTAACCAATTTCTC
PSH387	*pCambia1300-ProH1:H1G18IDR-eGFP*	TATGACCATGATTACGAATTCTCATT CTGTGATAGGGATGGAA
PSH411	*pCambia1300-ProH1:H1G18IDR-eGFP*	CCTCTGACATCGTCTTCTGAACTTAA GATCGGA
PSH412	*pCambia1300-ProH1:H1G18IDR-eGFP*	TCAGAAGACGATGTCAGAGGTGGAA ATAGAGAACG
PSH413	*pCambia1300-ProH1:H1G18IDR-eGFP*	CTCGCCCTTGCTCACGGATCCCAGA TTGAGAAGCAACAGCT

Supplemental Data. He et al. (2024). H1 condenses heterochromatin by phase separation. Plant Cell.

Supplemental Table S2. Protein sequences used for IDR prediction.

Species	UniProt code	Protein	Amino acid sequence
Arabidopsis thaliana	P26568	H1.1	MSEVEIENAATIEGNTAADAPVTDAAVEKKPA AKGRKTKNVKEVKEKKTVAAAPKKRTVSSHP TYEEMIKDAIVTLKERTGSSQYAIQKFIEEKRK ELPPTFRKLLLLNLKRLVASGKLVKVKASFKLP SASAKASSPKAAAEKSAPAKKKPATVAVTKAK RKVAAASKAKKTIAVKPKTAAAKKVTAKAKAK PVPRATAAATKRKAVDAKPKAKARPAKAAKT AKVTSPAKKAVAATKKVATVATKKKTPVKKVV KPKTVKSPAKRASSRVKK
Arabidopsis thaliana	P26569	H1.2	MSIEEENVPTTVDSGAADTTVKSPEKKPAAKG GKSKKTTTAKATKKPVKAAAPTKKKTTSSHPT YEEMIKDAIVTLKERTGSSQYAIQKFIEEKHKS LPPTFRKLLLVNLKRLVASEKLVKVKASFKIPS ARSAATPKPAAPVKKKATVVAKPKGKVAAAV APAKAKAAAKGTKKPAAKVVAKAKVTAKPKA KVTAAKPKSKSVAAVSKTKAVAAKPKAKERP AKASRTSTRTSPGKKVAAPAKKVAVTKKAPA KSVKVKSPAKRASTRKAKK
Bordetella pertussis	Q45370	H1	MATAKKAAKKAVKKPAAKKAAAKKATPAKKA AVKKVAVKKVAAKKPAVKKVAAKKPAAKKVA KKAVAKKAVAKKAVAKKAVAKKAVAKKAVAK KAVAKKAPAKKAVAKKAVAKKAVAKKAVAKK AVAKKAVPKKAPAKKAAPKKPATPPSTAAAP GAKTALNPAASWPVPDRRPSVTDLFAS
Chlamydophila psittaci	Q46204	HC1	MALKDTAKKMRDLLESIQRDLDKAERGNKAA AQRVRTDSIKLEKVAKVYRKESIKAEKSGLMT RKPATKAKKAAATKKAAPKPKIQAKAAPKAKA TTKKTPAKAKAKKSSKSRYLRK
Chlamydia trachomatis	Q46397	HC2	MLGVQKKRSTRKTAARKTVVRKPAAKKTAAK KAPVRKVAAKKTVARKTVAKKTVAARKPVAK KATAKKAPVRKAVAKKTVARKTVAKKTVAARK PVAKKATAKKAPVRKVAAKKTVARKTVAKKTV AARKPVAKKATAKKAPVRKAVAKKTVAKRVA STKKSSVAVKAGVCMKKHKHTAACGRVAAS GVKVCASAAKRKMNPNRSRTAHSWRQQLMK LVAR
Coxiella burnetii	Q45881	HQ1	MPAKKRKTTRQRRRSKARSASANTAALRKVS KERDQARRKLRAAQKKLAKAKKDASRKLAKL RKEAARKVAAAKKTRAPSKKGRKKATRKKGG GRSRKTARKVSTMKRGRGRPRKKA
